# A Therapeutic Non-self-reactive SARS-CoV-2 Antibody Protects from Lung Pathology in a COVID-19 Hamster Model

**DOI:** 10.1016/j.cell.2020.09.049

**Published:** 2020-11-12

**Authors:** Jakob Kreye, S. Momsen Reincke, Hans-Christian Kornau, Elisa Sánchez-Sendin, Victor Max Corman, Hejun Liu, Meng Yuan, Nicholas C. Wu, Xueyong Zhu, Chang-Chun D. Lee, Jakob Trimpert, Markus Höltje, Kristina Dietert, Laura Stöffler, Niels von Wardenburg, Scott van Hoof, Marie A. Homeyer, Julius Hoffmann, Azza Abdelgawad, Achim D. Gruber, Luca D. Bertzbach, Daria Vladimirova, Lucie Y. Li, Paula Charlotte Barthel, Karl Skriner, Andreas C. Hocke, Stefan Hippenstiel, Martin Witzenrath, Norbert Suttorp, Florian Kurth, Christiana Franke, Matthias Endres, Dietmar Schmitz, Lara Maria Jeworowski, Anja Richter, Marie Luisa Schmidt, Tatjana Schwarz, Marcel Alexander Müller, Christian Drosten, Daniel Wendisch, Leif E. Sander, Nikolaus Osterrieder, Ian A. Wilson, Harald Prüss

**Affiliations:** 1 German Center for Neurodegenerative Diseases (DZNE) Berlin, 10117 Berlin, Germany; 2 Helmholtz Innovation Lab BaoBab (Brain Antibody-omics and B-cell Lab), 10117 Berlin, Germany; 3 Department of Neurology and Experimental Neurology, Charité-Universitätsmedizin Berlin, Corporate Member of Freie Universität Berlin, Humboldt-Universität Berlin, and Berlin Institute of Health, 10117 Berlin, Germany; 4 Department of Pediatric Neurology, Charité-Universitätsmedizin Berlin, Corporate Member of Freie Universität Berlin, Humboldt-Universität Berlin, and Berlin Institute of Health, 10117 Berlin, Germany; 5 Berlin Institute of Health (BIH), 10178 Berlin, Germany; 6 Neuroscience Research Center (NWFZ), Cluster NeuroCure, Charité-Universitätsmedizin Berlin, Corporate Member of Freie Universität Berlin, Humboldt-Universität Berlin, and Berlin Institute of Health, 10117 Berlin, Germany; 7 Institute of Virology, Charité-Universitätsmedizin Berlin, Corporate Member of Freie Universität Berlin, Humboldt-Universität zu Berlin, and Berlin Institute of Health, 10117 Berlin, Germany, and German Centre for Infection Research (DZIF), 10117 Berlin, Germany; 8 Department of Integrative Structural and Computational Biology, The Scripps Research Institute, La Jolla, CA 92037, USA; 9 Institute of Virology, Freie Universität Berlin, 14163 Berlin, Germany; 10 Institute of Integrative Neuroanatomy Berlin, Charité-Universitätsmedizin Berlin, Corporate Member of Freie Universität Berlin, Humboldt-Universität zu Berlin, and Berlin Institute of Health, 10117 Berlin, Germany; 11 Institute of Veterinary Pathology, Freie Universität Berlin, 14163 Berlin, Germany; 12 Veterinary Centre for Resistance Research, Freie Universität Berlin, 14163 Berlin, Germany; 13 Department of Rheumatology and Clinical Immunology, Charité-Universitätsmedizin Berlin, Corporate Member of Freie Universität Berlin, Humboldt-Universität Berlin, and Berlin Institute of Health, 10117 Berlin, Germany; 14 Department of Infectious Diseases and Respiratory Medicine, Charité-Universitätsmedizin Berlin, Corporate Member of Freie Universität Berlin, Humboldt-Universität Berlin, and Berlin Institute of Health, 10117 Berlin, Germany; 15 Department of Tropical Medicine, Bernhard Nocht Institute for Tropical Medicine and I. Department of Medicine, University Medical Center Hamburg-Eppendorf, 20359 Hamburg, Germany; 16 Center for Stroke Research Berlin, Charité-Universitätsmedizin Berlin, Corporate Member of Freie Universität Berlin, Humboldt-Universität Berlin, and Berlin Institute of Health, 10117 Berlin, Germany; 17 Excellence Cluster NeuroCure Berlin, Charité-Universitätsmedizin Berlin, Corporate Member of Freie Universität Berlin, Humboldt-Universität Berlin, and Berlin Institute of Health, 10117 Berlin, Germany; 18 German Centre for Cardiovascular Research (DZHK), Partner Site Berlin, Charité-Universitätsmedizin Berlin, Corporate Member of Freie Universität Berlin, Humboldt-Universität Berlin, and Berlin Institute of Health, 10785 Berlin, Germany; 19 Department of Infectious Diseases and Public Health, Jockey Club College of Veterinary Medicine and Life Sciences, City University of Hong Kong, Hong Kong; 20 The Skaggs Institute for Chemical Biology, The Scripps Research Institute, La Jolla, CA 92037, USA

**Keywords:** COVID-19, SARS-CoV-2, monoclonal antibody, neutralizing antibody, crystal structures, autoreactivity, self-reactivity, self-antigens, hamster model, post-exposure

## Abstract

The emergence of SARS-CoV-2 led to pandemic spread of coronavirus disease 2019 (COVID-19), manifesting with respiratory symptoms and multi-organ dysfunction. Detailed characterization of virus-neutralizing antibodies and target epitopes is needed to understand COVID-19 pathophysiology and guide immunization strategies. Among 598 human monoclonal antibodies (mAbs) from 10 COVID-19 patients, we identified 40 strongly neutralizing mAbs. The most potent mAb, CV07-209, neutralized authentic SARS-CoV-2 with an IC_50_ value of 3.1 ng/mL. Crystal structures of two mAbs in complex with the SARS-CoV-2 receptor-binding domain at 2.55 and 2.70 Å revealed a direct block of ACE2 attachment. Interestingly, some of the near-germline SARS-CoV-2-neutralizing mAbs reacted with mammalian self-antigens. Prophylactic and therapeutic application of CV07-209 protected hamsters from SARS-CoV-2 infection, weight loss, and lung pathology. Our results show that non-self-reactive virus-neutralizing mAbs elicited during SARS-CoV-2 infection are a promising therapeutic strategy.

## Highlights

• Characterization of potent human monoclonal SARS-CoV-2-neutralizing antibodies
• Some SARS-CoV-2 antibodies reacted with mammalian self-antigens in different organs
• Crystal structures of two antibodies in complex with SARS-CoV-2 RBD at 2.55/2.70 Å
• Post-exposure antibody treatment protected from lung damage in infected hamsters

## Graphical Abstract

**Figure:**
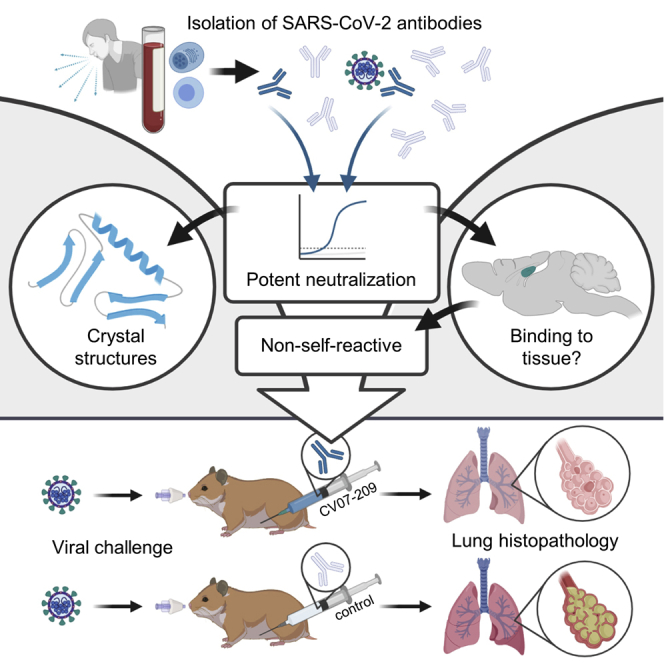

## Teaser

Kreye et al. report isolation and characterization of monoclonal antibodies from COVID-19 patients, some of which were found to display autoreactivity with mammalian self-antigens in different organs. Crystal structures of two antibodies in complex with the SARS-CoV-2 spike RBD reveal antibody engagement with the ACE2 binding site from different approach angles. One antibody was evaluated further for *in vivo* efficacy and found to be both protective and efficacious post-challenge in a hamster infection model.

## Introduction

Severe acute respiratory syndrome coronavirus 2 (SARS-CoV-2) started emerging in humans in late 2019 and rapidly became a pandemic with millions of cases worldwide. SARS-CoV-2 infection causes coronavirus disease 2019 (COVID-19) with severe respiratory symptoms, pathological inflammation, and multi-organ dysfunction, including acute respiratory distress syndrome, cardiovascular events, coagulopathies, and neurological symptoms (Helms et al., 2020; Zhou et al., 2020; Zhu et al., 2020). Some aspects of the diverse clinical manifestations may result from a hyperinflammatory response, as suggested by reduced mortality in hospitalized COVID-19 patients under dexamethasone therapy (Horby et al., 2020).

Understanding the immune response to SARS-CoV-2 is of utmost importance. Multiple recombinant SARS-CoV-2 monoclonal antibodies (mAbs) from convalescent patients have been reported (Brouwer et al., 2020; Cao et al., 2020; Ju et al., 2020; Kreer et al., 2020; Robbiani et al., 2020; Rogers et al., 2020; Wec et al., 2020). mAbs targeting the receptor-binding domain (RBD) of the viral spike protein S1 can compete with its binding to human angiotensin-converting enzyme 2 (ACE2) and prevent virus entry and subsequent replication (Cao et al., 2020; Ju et al., 2020; Walls et al., 2020). Potent virus-neutralizing mAbs that were isolated from diverse variable immunoglobulin (Ig) genes typically carry low levels of somatic hypermutations (SHMs). Several of these neutralizing mAbs selected for *in vitro* efficacy showed prophylactic or therapeutic potential in animal models (Cao et al., 2020; Liu et al., 2020; Rogers et al., 2020; Zost et al., 2020). The low number of SHMs suggests limited affinity maturation in germinal centers compatible with an acute infection. Near-germline mAbs usually constitute the first line of defense against pathogens but carry the risk of self-reactivity to autoantigens (Lerner, 2016; Liao et al., 2011; Zhou et al., 2007). Although critical for therapeutic use in humans, the potential tissue reactivity of near-germline SARS-CoV-2 antibodies has so far not been examined.

Here we systematically selected 18 strongly neutralizing mAbs of 598 antibodies from 10 COVID-19 patients by characterization of their biophysical properties, authentic SARS-CoV-2 neutralization, and exclusion of off-target binding to murine tissue. Additionally, we solved two crystal structures of neutralizing mAbs in complex with the RBD, showing antibody engagement with the ACE2 binding site from different approach angles. Finally, we selected mAb CV07-209 for *in vivo* evaluation because of its *in vitro* efficacy and absence of tissue reactivity. Systemic application of CV07-209 in a hamster model of SARS-CoV-2 infection led to a profound reduction of clinical, paraclinical, and histopathological COVID-19 pathology, reflecting its potential for translational application in patients with COVID-19.

## Results

### Antibody Repertoire Analysis of COVID-19 Patients

**Figure 1:**
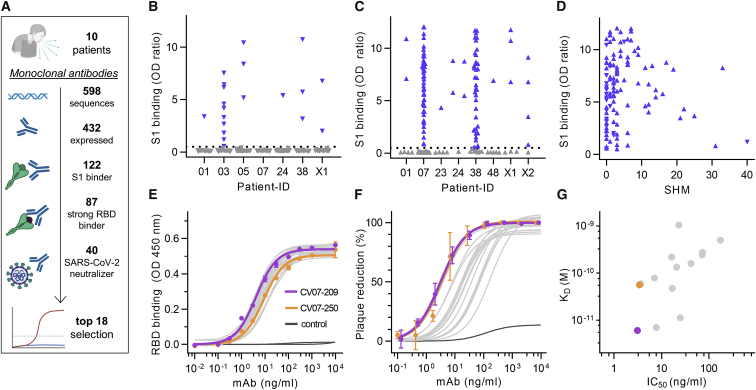
Identification and Characterization of Potent SARS-CoV-2-Neutralizing mAbs (A) Diagram depicting the strategy for isolation of 18 potently neutralizing mAbs (top 18). (B) Normalized binding to S1 of SARS-CoV-2 for mAbs isolated from ASCs (inverted triangles; blue, S1-binding; gray, not S1-binding). OD, optical density in ELISA. (C) Normalized binding to S1 of SARS-CoV-2 for mAbs isolated from S1-stained MBCs (triangles; colors as in B). (D) S1-binding plotted against the number of somatic hypermutations (SHMs) for all S1-reactive mAbs. (E) Concentration-dependent binding of the top 18 SARS-CoV-2 mAbs to the RBD of S1 (mean ± SD from two wells of one experiment). (F) Concentration-dependent neutralization of authentic SARS-CoV-2 plaque formation by the top 18 mAbs (mean ± SD from two independent measurements). (G) Apparent affinities of mAbs to RBDs (K_D_ determined by surface plasmon resonance) plotted against IC_50_ of authentic SARS-CoV-2 neutralization. See also Figures S1, S2, S3, S4, and S5 and Tables S1, S2, and S3.

**Figure S1:**
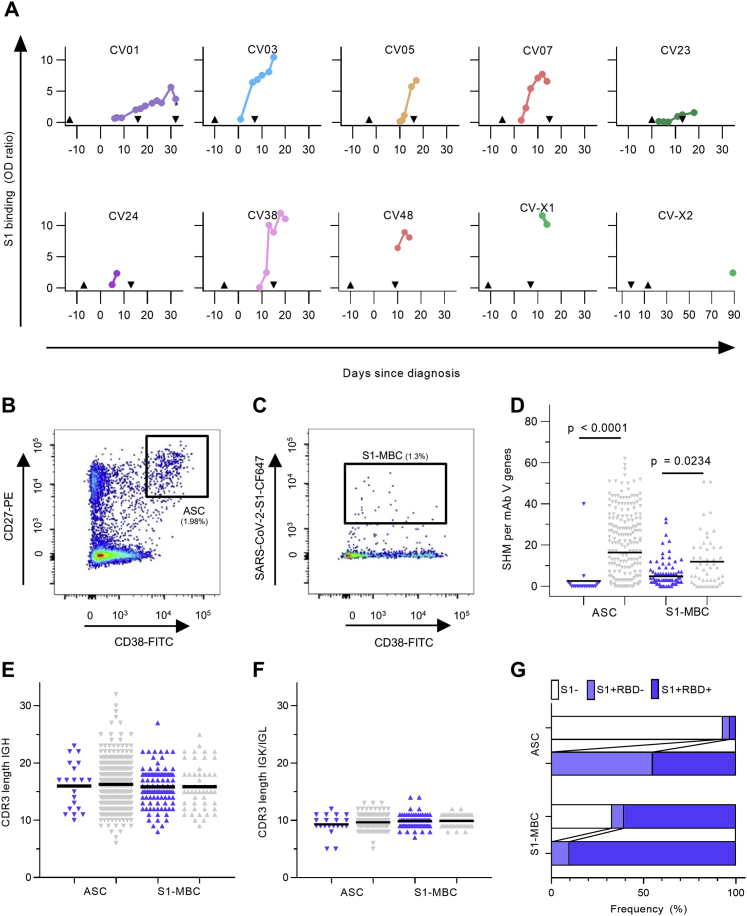
SARS-CoV-2-S1 Serum IgG Response from COVID-19 Patients, Flow Cytometry Gating, and Characteristics of Ig Sequences, Related to Figure 1 and Tables S1 and S2 (A) Serum IgG response determined as the normalized optical density (OD) in a SARS-CoV-2-S1 ELISA in relation to the time point of diagnosis defined by the first positive qPCR test. Upward arrowhead denotes the appearance of first symptoms. Downward arrowhead denotes the PBMC isolation. From patient CV01, PBMC samples were isolated at two time points as indicated by the second downward arrow with an asterisk (^∗^). (B-C) A representative flow cytometry plot from patient CV38 indicating gating on (B) CD19^+^CD27^+^antibody-secreting cells (ASC) and (C) SARS-CoV-2-S1-stained memory B cells (S1-MBC). Cells were pre-gated on live CD19^+^ B cells. (D) Comparison of somatic hypermutation (SHM) count within immunoglobulint V genes combined from heavy and light chains of S1-reactive (S1+, blue) and non-S1-reactive (S1-, gray) mAbs. Statistical significance was determined using a Kruskal-Wallis test with Dunn’s multiple comparison test. (ASC: n = 20 S1+, n = 260 S1-; S1-MBC: n = 102 S1+, n = 50 S1-, n-values represent number of mAbs). All expressed mAbs are displayed. Each triangle represents one mAb, isolated from an ASC (inverted triangle) or a S1-MBC (triangle). Bars indicate mean. (E-F) Length comparison of complementarity-determining region (CDR) 3 amino acid sequences between S1+ and S1- mAbs within (E) heavy and (F) light chains. Bars indicate mean. Symbols and colors have the same meaning as in (D). (G) Frequency of RBD-binder (S1+RBD+) and non-RBD-binder (S1+RBD-) relative to all expressed mAbs (upper lanes) and relative to S1+ mAbs (lower lanes).

We first characterized the B cell response in COVID-19 using single-cell Ig gene sequencing of human mAbs (Figure 1A). From 10 COVID-19 patients with serum antibodies to the S1 subunit of the SARS-CoV-2 spike protein (Figure S1A; Table S1), we isolated two populations of single cells from peripheral blood mononuclear cells with fluorescence-activated cell sorting (FACS): CD19^+^CD27^+^CD38^+^ antibody-secreting cells (ASCs) reflecting the overall humoral immune response and SARS-CoV-2-S1-labeled CD19^+^CD27^+^ memory B cells (S1-MBCs) for characterization of antigen-specific responses (Figures S1B and S1C). We obtained 598 functional paired heavy- and light-chain Ig sequences (Table S2). Of 432 recombinantly expressed mAbs, 122 were reactive to SARS-CoV-2-S1 (S1+) with a frequency of 0.0%–18.2% (median, 7.1%) within ASCs and 16.7%–84.1% (median, 67.1%) within S1-MBCs (Figures 1B and 1C). Binding to S1 did not depend on affinity maturation, as measured by the number of SHMs (Figure 1D). Compared with mAbs not reactive to SARS-CoV-2-S1, S1+ mAbs had fewer SHMs but equal lengths of their light- and heavy-chain complementarity-determining region 3 (CDR3) (Figures S1D–S1F). Within the ASC and S1-MBC population, 45.0% and 90.2% of S1+ mAbs, respectively, bound the RBD (Figure S1G).

**Figure S2:**
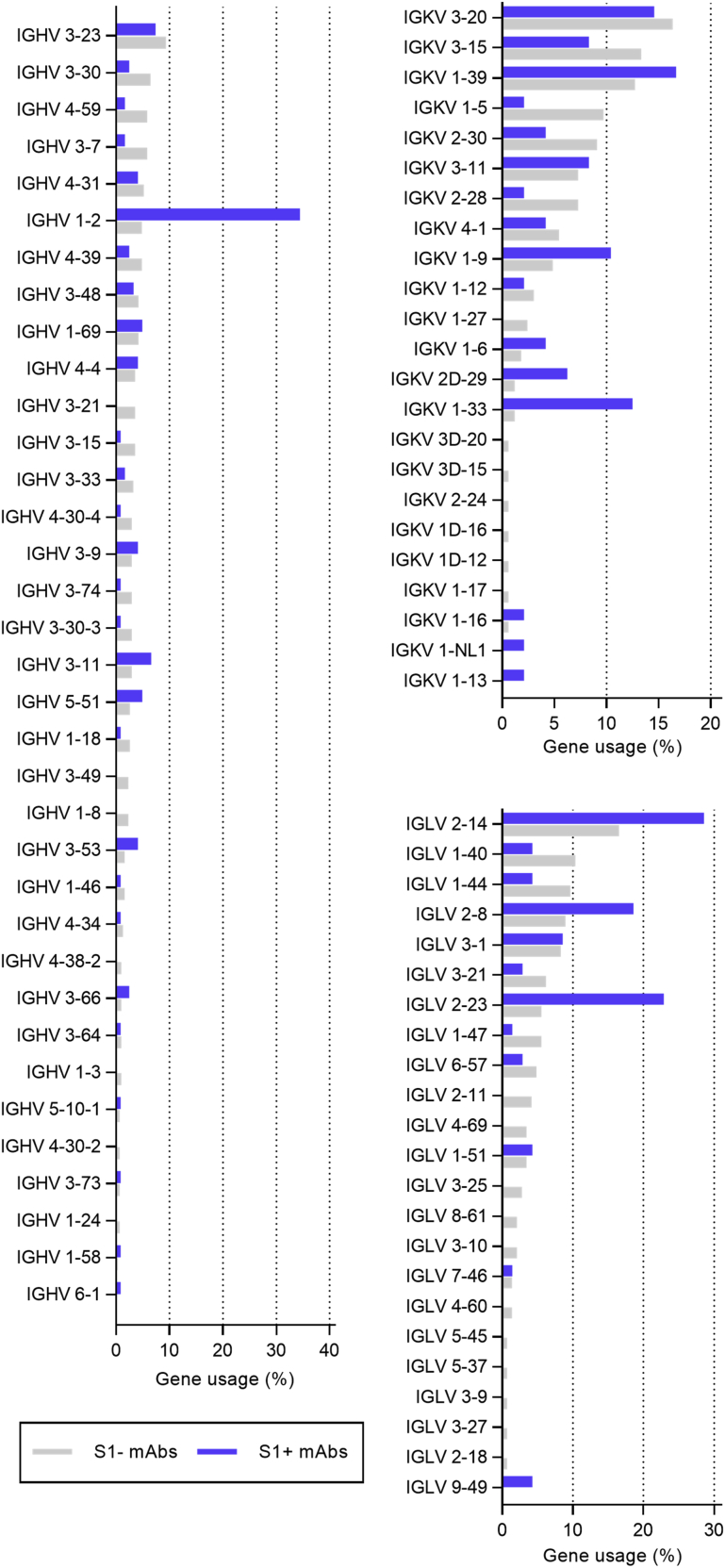
Comparison of Variable Gene Use, Related to Figure 1 and Table S2 Comparison of gene usage between SARS-CoV-2-S1-reactive (S1+) and non-reactive (S1-) mAbs is shown for immunoglobulin (A) variable heavy (IGHV), (B) variable kappa (IGKV) and (C) variable lambda (IGLV) genes. Bars depict percentage of gene usage of all expressed mAbs within each group.

**Figure S3:**
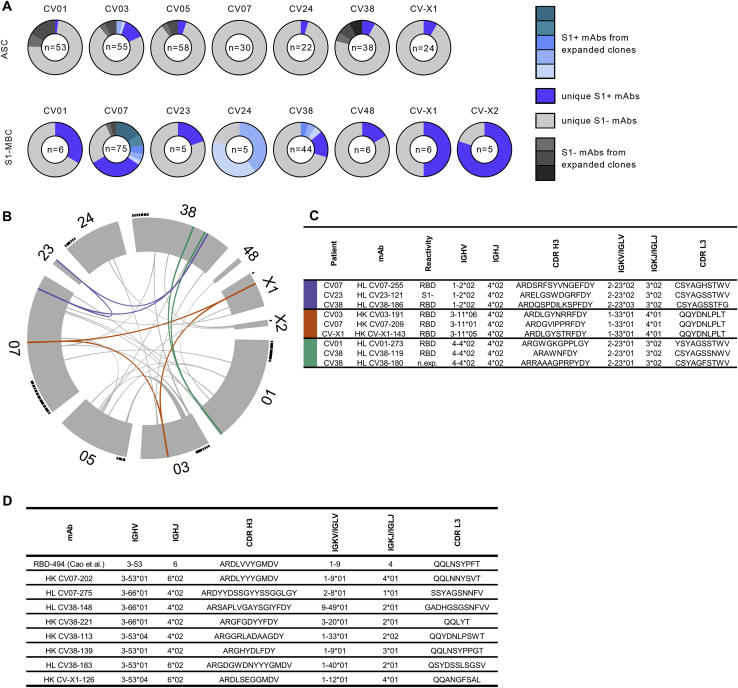
Clonal Expansion and Public or Common Clonotypes, Related to Figure 1 and Table S2. (A) Pie charts represent clonal relationship of all expressed mAbs from each donor separately for antibody secreting cells (ASC) and S1-stained memory B cells (S1-MBC). mAbs were considered S1-reactive (S1+) or non-S1-reactive (S1-) based on SARS-CoV-2-S1 ELISA measurements. Antibodies were considered to be clonally expanded when they were isolated from multiple cells. (B) Circos plot displays all isolated mAbs from ten donors. Interconnecting lines indicate relationship between mAbs that share the same V and J gene on both Ig heavy and light chain. Such public or shared clonotypes in which more than 50% of mAbs are S1-reactive are represented as colored lines. Small black angles at the outer circle border indicate expanded clones within the respective donor. (C) Properties of public clonotypes from S1+ mAbs according to the colors used in (B) with sequence similarities between mAbs isolated from different donors, also within CDR3. (D) Public or common antibody response using VH3-53 and VH3-66 genes. IGHV, IGHJ IGKV, IGKJ, IGLV, IGLJ = V (variable) and J (joining) genes of immunoglobulin heavy, kappa, lambda chains; CDR = complementarity-determining region; n.exp. = not expressed.

S1+ mAbs were enriched in certain Ig genes, including variable heavy (VH)1-2, VH3-53, VH3-66, variable kappa (VK)1-33, and variable lambda (VL)2-14 (Figure S2). We identified clonally related antibody clones within patients and public and shared S1+ clonotypes from multiple patients (Figures S3A and S3B). Some public or shared clonotypes had been reported previously, such as IGHV3-53 and IGHV3-66 (Figure S3D; Cao et al., 2020; Yuan et al., 2020a), whereas others were newly identified, such as IGHV3-11 (Figure S3C).

### Identification and Characterization of Potent SARS-CoV-2-Neutralizing mAbs

**Figure S4:**
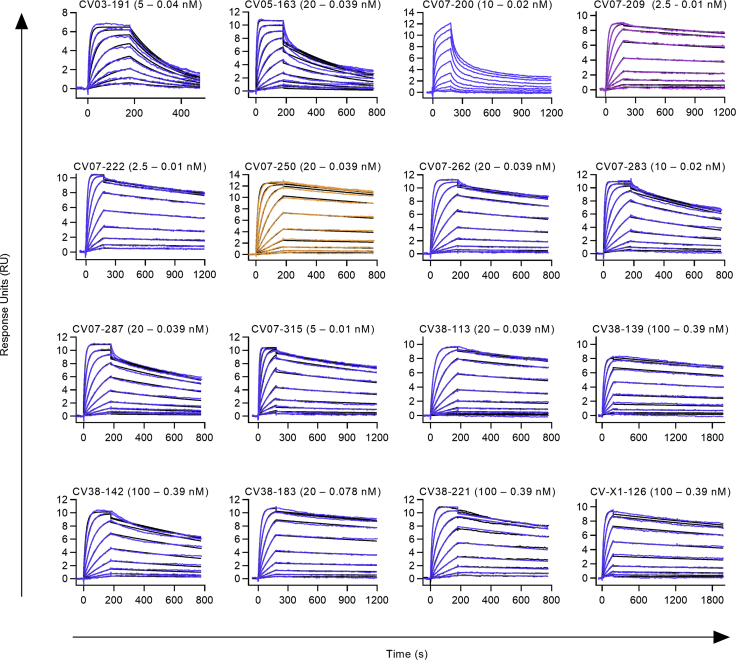
Binding Kinetic Measurements of mAbs to the RBD, Related to Figure 1 and Table S3 Binding kinetics of mAbs to RBD were modeled (black) from multi-cycle surface plasmon resonance (SPR) measurements (blue, purple, orange). Fitted monovalent analyte model is shown. For CV07-200, neither a bivalent nor a monovalent analyte model described the data accurately (no model is shown). Three out of the 18 selected mAbs for detailed characterization (top 18) were not analyzed using multi-cycle-kinetics: CV07-270 was excluded as it interacted with the anti-mouse IgG reference surface on initial qualitative measurements. CV07-255 and CV-X2-106 were not analyzed since they showed biphasic binding kinetics and relatively fast dissociation rates in initial qualitative measurements. Non-neutralizing CV03-191, a mAb not included in the top 18 mAbs, was included in the multicycle experiments as it has the same clonotype as strongly neutralizing CV07-209 (Figure S4C). All measurements are performed by using a serial 2-fold dilution of mAbs on reversibly immobilized SARS-CoV-2-S1 RBD-mFc.

**Figure S5:**
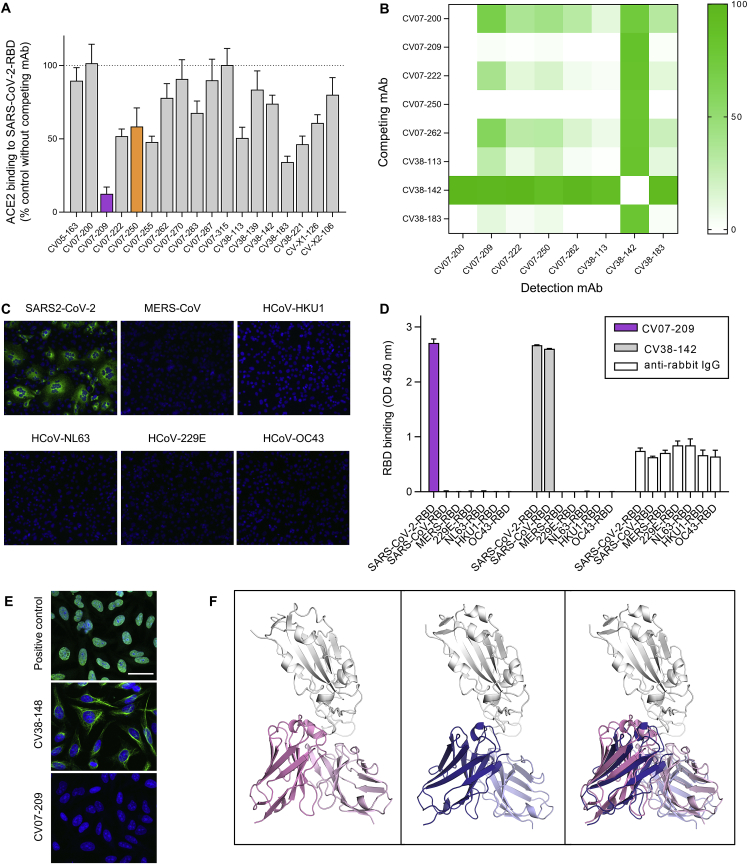
Binding Epitope Characterization of Selected mAbs, Related to Figures 1, 3, and 4 and Table S3 (A) Competition for RBD binding between top 18 mAbs and ACE2. ELISA-based measurements of human ACE2 binding to SARS-CoV-2 RBD after pre-incubation with the indicated neutralizing mAbs. Values are shown relative to antibody-free condition as mean + SD from three independent measurements. (B) Competition for RBD binding between combinations of potent neutralizing mAbs is illustrated as a heatmap. Shades of green indicate the degree of competition for RBD binding of detection mAb in presence of 100-fold excess of competing mAb relative to non-competition conditions. Green squares indicate no competition. Values are shown as mean of two independent experiments. (C) Representative immunofluorescence staining on VeroB4 cells overexpressing spike protein of indicated coronavirus with SARS-CoV-2 mAb CV07-209 at 5 μg/ml. For all other 17 of the selected 18 mAbs (top 18, Table S3), similar results were obtained. (D) Binding of indicated mAbs to fusion proteins containing the RBD of indicated coronaviruses and the constant region of rabbit IgG revealed by ELISA. For all other top 18 mAbs, similar results were obtained as for CV07-209. Values indicate mean + SD from two wells of one experiment. (E) Representative HEp-2 cell staining with a commercial anti-nuclear antibody as positive control revealed nuclear binding (top). S1-reactive non-neutralizing mAb CV38-148 exhibited cytoplasmatic binding (middle). Neutralizing mAb CV07-209 showed no binding (bottom). All mAbs selected for detailed characterization (top 18, Table S3) revealed similar results like CV07-209 when used at 50 μg/ml. Representative scale bar: 25 μm. (F) Structural comparison of CV07-270/RBD and P2B-2F6/RBD complexes. Structure of CV07-270 (pink, left) and structure of P2B-2F6 (PDB 7BWJ) (Ju et al., 2020) (blue, middle) in complex with RBD (white), as well as superimposition of the structures of CV07-270/RBD and P2B-2F6/RBD based on the RBD (right).

We next determined mAbs with the highest capacity to neutralize SARS-CoV-2 in plaque reduction neutralization tests (PRNTs) using an authentic virus (Munich isolate 984) (Wölfel et al., 2020). Of 87 mAbs strongly binding to the RBD, 40 showed virus neutralization with a half-maximal inhibitory concentration (IC_50_) of 250 ng/mL or less and were considered neutralizing antibodies (Figure 1A; Table S2), of which 18 (top 18) were selected for further characterization (Table S3). The antibodies bound to the RBD with a half-maximal effective concentration (EC_50_) of 3.8–14.2 ng/mL (Figure 1E) and an equilibrium dissociation constant (K_D_) of 6.0 pM to 1.1 nM (Figure S4; Table S3), neutralizing SARS-CoV-2 with an IC_50_ value of 3.1–172 ng/mL (Figure 1F; Table S3). The antibody with the highest apparent affinity, CV07-209, was also the strongest neutralizer (Figure 1G). We hypothesized that the differences in neutralizing capacity relate to different interactions with the ACE2 binding site. Indeed, the strongest neutralizing mAbs, CV07-209 and CV07-250, reduced ACE2 binding to the RBD to 12.4% and 58.3%, respectively. Other top 18 mAbs, including CV07-270, interfered only weakly with ACE2 binding (Figure S5A).

The spike proteins of SARS-CoV-2 and SARS-CoV share more than 70% amino acid sequence identity, whereas sequence identity between SARS-CoV-2 and MERS-CoV and other endemic coronaviruses is significantly lower (Barnes et al., 2020). To analyze potential cross-reactivity of mAbs to other coronaviruses, we tested for binding of the top 18 mAbs to the RBD of SARS-CoV, MERS-CoV, and the human endemic coronaviruses 229E, NL63, HKU1, and OC43. CV38-142 detected the RBDs of SARS-CoV-2 and SARS-CoV, whereas no other mAb was cross-reactive to additional coronaviruses (Figures S5C and S5D). To further characterize the epitope of neutralizing mAbs, we performed ELISA-based epitope binning experiments using biotinylated antibodies. Co-application of paired mAbs showed competition of most neutralizing antibodies for RBD binding (Figure S5B). As an exception, the SARS-CoV cross-reactive CV38-142 bound the RBD irrespective of the presence of other mAbs, suggesting an independent and conserved target epitope (Figure S5B).

### Near-Germline SARS-CoV-2 Neutralizing Antibodies Can Bind to Murine Tissue

Many SARS-CoV-2-neutralizing mAbs carry few SHMs or are in germline configuration (Figure 1D; Ju et al., 2020; Kreer et al., 2020). Antibodies close to the germline might be reactive to more than one target (Zhou et al., 2007). Prompted by the abundance of near-germline SARS-CoV-2 antibodies and to exclude potential side effects of mAb treatment, we next analyzed whether SARS-CoV-2 antibodies can bind to self-antigens.

**Figure 2:**
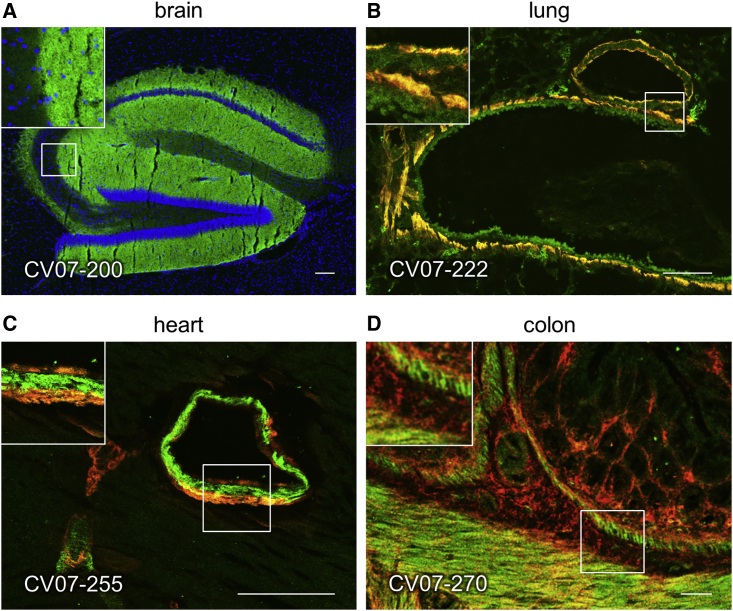
SARS-CoV-2-Neutralizing Antibodies Can Bind to Murine Tissue Immunofluorescence staining of SARS-CoV-2 mAbs (green) on murine organ sections showed specific binding to distinct anatomical structures. (A) Staining of hippocampal neuropil with CV07-200 (cell nuclei depicted in blue). (B) Staining of bronchial walls with CV07-222. (C) Staining of vascular walls with CV07-255. (D) Staining of intestinal walls with CV07-270. Smooth muscle tissue in (B)–(D) was co-stained with a commercial smooth muscle actin antibody (red). Scale bars, 100 μm. See also Table S3.

We tested binding of S1 mAbs to unfixed murine tissues. Surprisingly, four of the top 18 potent SARS-CoV-2-neutralizing mAbs showed anatomically distinct tissue reactivities (Figure 2; Table S3). CV07-200 intensively stained brain sections in the hippocampal formation, olfactory bulb, cerebral cortex, and basal ganglia (Figure 2A). CV07-222 also bound to brain tissue as well as to smooth muscle (Figure 2B). CV07-255 and CV07-270 were reactive to smooth muscle from sections of lung, heart, kidney, and colon but not liver (Figures 2C and 2D; Table S3). None of the top 18 mAbs bound to HEp-2 cells, cardiolipin, or beta-2 microglobulin as established polyreactivity-related antigens (Jardine et al., 2016; Figure S5E).

### Crystal Structures of Two mAbs Approaching the ACE2 Binding Site from Different Angles

**Figure 3:**
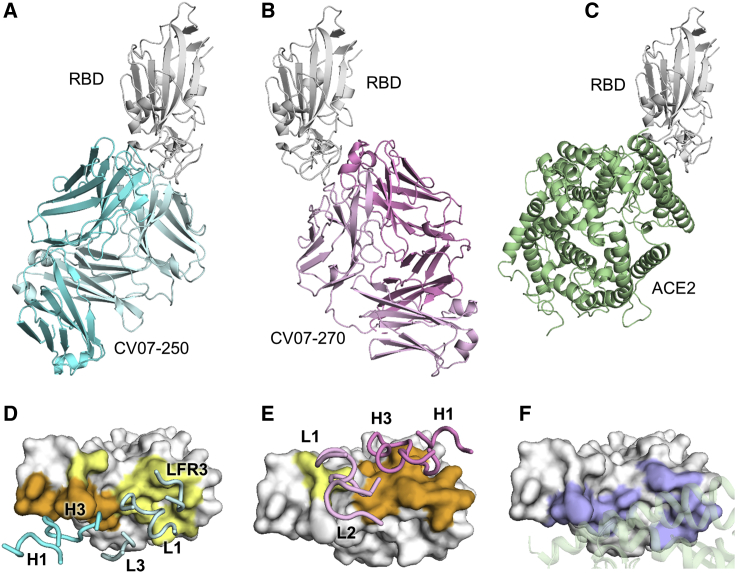
Crystal Structures of mAbs in Complex with the SARS-CoV-2 RBD (A) CV07-250 (cyan) in complex with the RBD (white). (B) CV07-270 (pink) in complex with the RBD (white). (C) Human ACE2 with the SARS-CoV-2 RBD (PDB: 6M0J; Lan et al., 2020). (D and E) Epitopes of (D) CV07-250 and (E) CV07-270. Epitope residues contacting the heavy chain are shown in orange and those contacting the light chain in yellow. CDR loops and the framework region that contact the RBD are labeled. (F) ACE2-binding residues on the RBD (blue) in the same view as in (D) and (E). The ACE2-interacting region is shown in green within a semi-transparent cartoon representation. See also Figures S5 and S6 and Tables S4 and S5.

Diffraction-quality crystals were obtained for the SARS-CoV-2 RBD complexed with two individual neutralizing mAbs, CV07-250 and CV07-270, which have notable differences in the number of SHMs, extent of ACE2 competition, and binding to murine tissue. CV07-250 (IC_50_ = 3.5 ng/mL) had 33 SHMs (17/16 on the heavy and light chain, respectively) and strongly reduced ACE2 binding and showed no binding to murine tissue. In contrast, CV07-270 (IC_50_ = 82.3 ng/mL) had only 2 SHMs (2/0), did not reduce ACE2 binding in our assay, and showed binding to smooth muscle tissue. Using X-ray crystallography, we determined the structures of CV07-250 and CV07-270 in complex with the SARS-CoV-2 RBD to resolutions of 2.55 and 2.70 Å, respectively (Figure 3; Tables S4 and S5).

**Figure 4:**
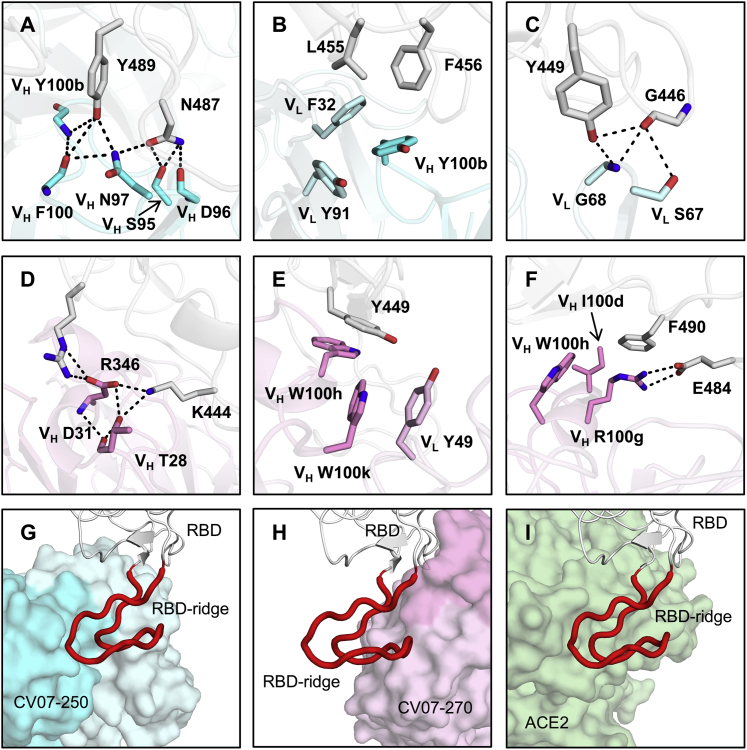
Interactions and Angle of Approach at the RBD-Antibody Interface (A–C) Key interactions between CV07-250 (cyan) and the RBD (white) are highlighted. (A) CDR H3 of CV07-250 forms a hydrogen bond network with RBD Y489 and N487. (B) VH Y100b (CDR H3), VL F32 (CDR L1), and VL Y91 (CDR L3) of CV07-250 form a hydrophobic aromatic patch for interaction with RBD L455 and F456. (C) The side chain of VL S67 and backbone amide of VL G68 from FR3 are engaged in a hydrogen bond network with RBD G446 and Y449. (D–F) Interactions between CV07-270 (cyan) and the RBD (white). (D) Residues in CDR H1 of CV07-270 participate in an electrostatic and hydrogen bond network with RBD R346 and K444. (E) VH W100h and VH W100k on CDR H3 of CV07-270 make π-π stacking interactions with Y449. VH W100k is also stabilized by a π-π stacking interaction with VL Y49. (F) VH R100 g on CDR H3 of CV07-270 forms an electrostatic interaction with RBD E484 as well as a π-cation interaction with RBD F490. Oxygen atoms are shown in red and nitrogen atoms in blue. Hydrogen bonds are represented by dashed lines. (G–I) Magnified views of the different RBD ridge interactions with (G) CV07-250, (H) CV07-270, and (I) ACE2 (PDB: 6M0J; Lan et al., 2020). The ACE2-binding ridge in the RBD is represented by a backbone ribbon trace in red. See also Figures S5 and S6 and Tables S4 and S5.

The binding mode of CV07-250 to the RBD is unusual in that it is dominated by the light chain (Figures 3A and 3D), whereas in CV07-270, the heavy chain dominates, as found frequently in other antibodies (Figures 3B and 3E). Upon interaction with the RBD, CV07-250 has a buried surface area (BSA) of 399 Å^2^ and 559 Å^2^ on the heavy and light chains, respectively, compared with 714 Å^2^ and 111 Å^2^ in CV07-270. CV07-250 uses CDR H1, H3, L1, and L3 and framework region 3 (LFR3) for RBD interaction (Figures 3D and 4A–4C), whereas CV07-270 interacts with CDR H1, H3, L1, and L2 (Figures 3E and 4D–4F).

The epitope of CV07-250 completely overlaps with the ACE2 binding site with a similar angle of approach as ACE2 (Figures 3A, 3C, 4G, and 4I). In contrast, the CV07-270 epitope only partially overlaps with the ACE2 binding site, and the antibody approaches the RBD from a different angle compared with CV07-250 and ACE2 (Figures 3B, 3C, 4H, and 4I), explaining differences in ACE2 competition. Although CV07-250 and CV07-270 contact 25 epitope residues, only seven residues are shared (G446/G447/E484/G485/Q493/S494/Q498). Furthermore, CV07-270 binds to a similar epitope as the SARS-CoV-2-neutralizing antibody P2B-2F6 (Ju et al., 2020) with a similar angle of approach (Figure S5F). In fact, 18 of 20 residues in the P2B-2F6 epitope overlap with the CV07-270 epitope, although CV07-270 and P2B-2F6 are encoded by different germline genes for the heavy and light chains. Thus, these two mAbs represent antibodies encoded by different germline genes that bind to the same epitope in the RBD with near-identical binding modes and approach angles. This structural convergence is also encouraging for targeting this highly immunogenic epitope for vaccine development.

**Figure S6:**
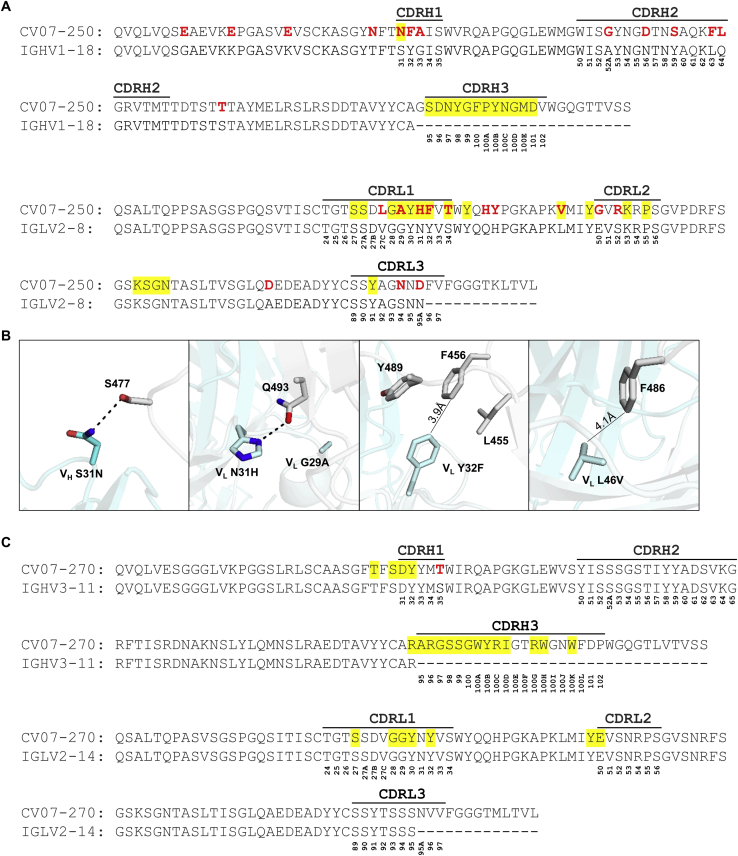
Comparison of Sequences of CV07-250 and CV07-270 with Their Putative Germline Sequences, Related to Figures 3 and 4 (A) Alignment of CV07-250 with the germline IGHV1-18 sequence (nucleotide SHM rate 5.8%) and IGLV2-8 (nucleotide SHM rate 5.4%). (B) Somatic mutations VH S31H, VL G29A, VL N31H, VL Y32F, VL S34T, and VL L46V are located in the CV07-250 paratope with other somatic mutations in all of the CDRs that may affect overall CDR conformation and interactions. Hydrogen bonds are represented by dashed lines. Distances between atoms are shown in solid lines. CV07-250 heavy chain is in dark cyan and light chain is in light cyan. SARS-CoV-2 RBD is in light gray. (C) Alignment of CV07-270 with the germline IGHV3-11 sequence (nucleotide SHM rate 0.7%) and IGLV2-14 (nucleotide SHM rate 0%). The regions that correspond to CDR H1, H2, H3, L1, L2, and L3 are indicated. Residues that differ from the germline are highlighted in red. Residues that interact with the RBD are highlighted in yellow. Residue positions in the CDRs are labeled according to the Kabat numbering scheme.

Interestingly, CV07-250 was isolated 19 days after symptom onset but had already acquired 33 SHMs, the highest number among all S1+ MBCs (Figure S1D). Some non-germline amino acids are not directly involved in RBD binding, including all five SHMs on CDR H2 (Figure S6). This observation suggests that CV07-250 could have been initially affinity matured against a different antigen.

### Prophylactic and Therapeutic mAbs in a COVID-19 Animal Model

**Figure 5:**
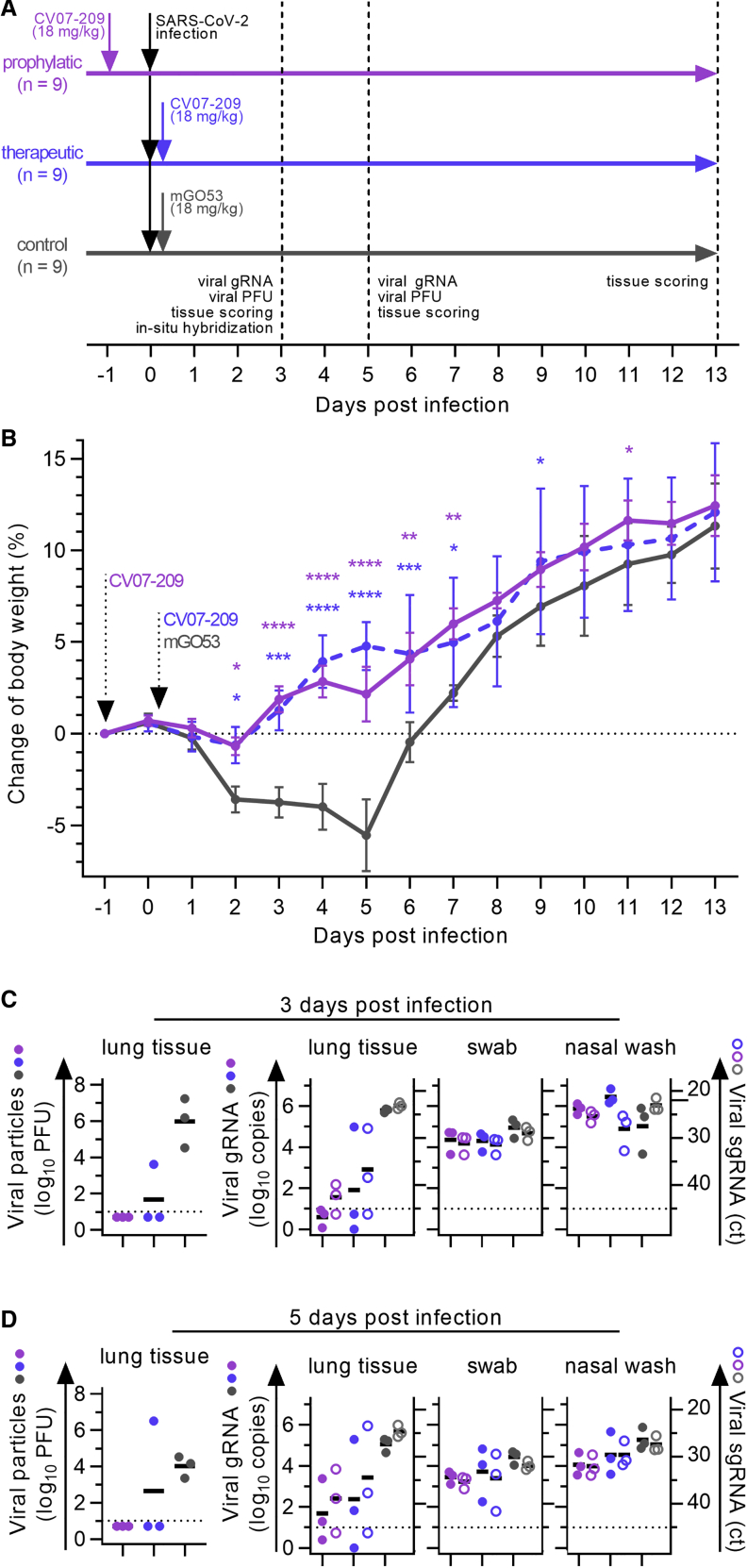
Prophylactic and Therapeutic Application of mAb CV07-209 in a COVID-19 Hamster Model (A) Schematic overview of the animal experiment. (B) Body weight of hamsters after virus challenge and prophylactic (pink) or therapeutic (blue) application of the SARS-CoV-2-neutralizing mAb CV07-209 or control antibody (mean ± SEM from 9 animals per group from days −1 to 3, n = 6 from days 4–5; n = 3 from days 6–13; mixed-effects model with post hoc Dunnett’s multiple tests in comparison with the control group; significance levels are shown as ^∗^p < 0.05, ^∗∗^p < 0.01, ^∗∗∗^p < 0.001, and ^∗∗∗∗^p < 0.0001 or not shown when not significant. (C and D) Left: quantification of plaque-forming units (PFU) from lung homogenates. Right: quantification of genomic SARS-CoV-2 RNA (gRNA) as copies per 10^5^ cellular transcripts (left y axis, filled circles) and cycle threshold (ct) of subgenomic SARS-CoV-2 RNA (sgRNA) detection (right y axis, unfilled circles) from samples and time points as indicated. Values for PFUs were set to 5 when not detected, gRNA copies below 1 were set to 1, and the ct of sgRNA was set to 46 when not detected. Bars indicate the mean. Dotted lines represent the detection threshold. See also Figure 6 and Table S6.

Next we selected mAb CV07-209 for evaluation of *in vivo* efficacy based on its high capacity to neutralize SARS-CoV-2 and the absence of reactivity to mammalian tissue. We used the hamster model of COVID-19 because it is characterized by rapid weight loss and severe lung pathology (Osterrieder et al., 2020). In this experimental setup, hamsters were infected intranasally with authentic SARS-CoV-2. Nine hamsters per group received a prophylactic application of CV07-209 24 h before virus challenge or a therapeutic application of CV07-209 or the control antibody mGO53 2 h after virus challenge (Figure 5A).

Hamsters under control mAb treatment lost 5.5% ± 4.4% (mean ± SD) of body weight, whereas those that received mAb CV07-209 as a therapeutic or prophylactic single dose gained 2.2% ± 3.4% or 4.8% ± 3.4% weight after 5 days post-infection (dpi), respectively. Mean body weights gradually converged in animals followed up until 13 dpi, reflecting recovery of control-treated hamsters from SARS-CoV-2 infection (Figure 5B).

To investigate the presence of SARS-CoV-2 in the lungs, we measured functional SARS-CoV-2 particles from lung tissue homogenates. Plaque-forming units were below the detection threshold for all animals in the prophylactic group and in 2 of 3 in the treatment group at 3 and 5 dpi (Figures 5C and 5D). qPCR measurements of lung viral genomic RNA copies revealed a 4–5 and 3–4 log reduction at both time points in the prophylactic and therapeutic groups, indicating a drastic decrease in SARS-CoV-2 particles in the lungs after CV07-209 application. Reduced virus replication and cell infection was confirmed by lowered detection of subgenomic viral RNA (Figures 5C and 5D). However, genomic and subgenomic RNA levels from nasal washes and laryngeal swaps were similar between all groups, indicating virus replication in the upper airways (Figures 5C and 5D).

**Figure 6:**
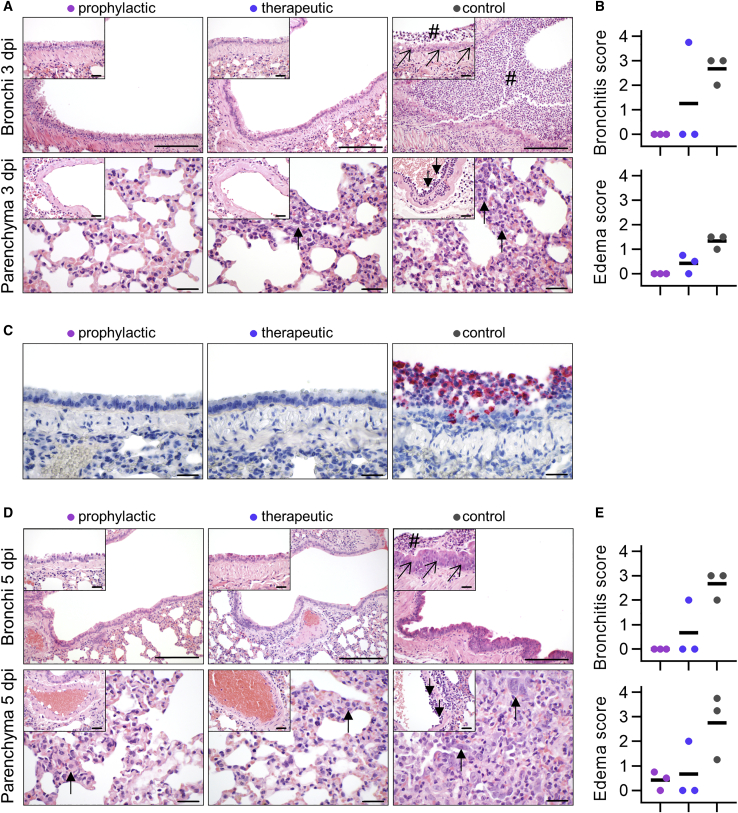
Histopathological Analysis of Hamsters after SARS-CoV-2 Infection (A) Histopathology of representative hematoxylin-and-eosin-stained, paraffin-embedded bronchi with inserted epithelium (top row) and lung parenchyma with inserted blood vessels (bottom row) at 3 dpi. Severe suppurative bronchitis with immune cell infiltration (hash symbol) is apparent only in the control-treated animals with necrosis of bronchial epithelial cells (diagonal arrows). Necro-suppurative interstitial pneumonia (upward arrows) with endothelialitis (downward arrows) is prominent in control-treated animals. Scale bars, 200 μm in the bronchus overview, 50 μm in all others. (B) Bronchitis and edema score at 3 dpi. Bars indicate the mean. (C) Detection of viral RNA (red) using *in situ* hybridization of representative bronchial epithelium present only in the control group. Scale bars, 50 μm. (D) Histopathology of representative lung sections from areas comparable with (A) at 5 dpi. Staining of bronchi of control-treated animals showed marked bronchial hyperplasia with hyperplasia of epithelial cells (diagonal arrow) and still existing bronchitis (hash symbol), absent in all prophylactically treated and in 2/3 therapeutically treated animals (top row). Lung parenchyma staining of control-treated animals showed severe interstitial pneumonia with marked type II alveolar epithelial cell hyperplasia and endothelialitis (insets, downward arrows). Compared with control-treated animals, prophylactically treated animals showed only mild signs of interstitial pneumonia with mild type II alveolar epithelial cell hyperplasia (upward arrow), whereas therapeutically treated animals showed a more heterogeneous picture, with 1/3 animals showing no signs of lung pathology, 1/3 animals showing only mild signs of interstitial pneumonia, and 1/3 animals showing moderate multifocal interstitial pneumonia. Scale bars, 200 μm in the bronchus overview, 50 μm in all others. (E) Bronchitis and edema score at 5 dpi. Bars indicate the mean. See also Figure 5 and Table S6.

Additionally, we performed histopathological analyses of infected hamsters. As expected, all lungs from control-treated animals sacrificed at 3 dpi revealed typical histopathological signs of necro-suppurative pneumonia with suppurative bronchitis, necrosis of bronchial epithelial cells, and endothelialitis (Figure 6A). At 5 dpi, control-treated animals showed marked bronchial hyperplasia, severe interstitial pneumonia with marked type II alveolar epithelial cell hyperplasia, and endothelialitis (Figure 6D). In contrast, animals receiving prophylactic treatment with CV07-209 showed no signs of pneumonia, bronchitis, necrosis of bronchial epithelial cells, or endothelialitis at 3 dpi. Mild interstitial pneumonia with mild type II alveolar epithelial cell hyperplasia became apparent 5 dpi. Animals receiving therapeutic CV07-209 treatment also showed a marked reduction in histopathological signs of COVID-19 pathology, although, at both time points, one of three animals showed mild bronchopulmonary pathology with signs of interstitial pneumonia and endothelialitis. These qualitative findings were mirrored in the reduction of bronchitis and edema scores (Figures 6B and 6E; Table S6).

To confirm the absence of viral particles under CV07-209 treatment, we performed *in situ* hybridization of viral RNA at 3 dpi. No viral RNA was detectable in the prophylactic group, whereas all animals in the control group and one in the therapeutic group revealed intensive staining of viral RNA in proximity of bronchial epithelial cells (Figure 6C). These findings show that systemic application of the SARS-CoV-2-neutralizing mAb CV07-209 protects hamsters from COVID-19 lung pathology and weight loss in prophylactic and therapeutic settings.

## Discussion

Driven by the pandemic spread of COVID-19 in early 2020, numerous groups have reported isolation, characterization, structural analysis, and animal model application of SARS-CoV-2-neutralizing mAbs (Barnes et al., 2020; Brouwer et al., 2020; Cao et al., 2020; Ju et al., 2020; Kreer et al., 2020; Robbiani et al., 2020; Rogers et al., 2020; Wec et al., 2020). In many places, our work confirms previous results, including observation of a shared antibody response against the SARS-CoV-2 spike protein, identification of ACE2 blocking as an important mechanism of virus neutralization, isolation of high-affinity near-germline antibodies, and *in vivo* efficacy of prophylactic mAb application. Our results add several findings to the growing knowledge about the humoral immune response in SARS-CoV-2 infection.

First, we provide two structures of neutralizing mAbs identified in this study as binding to the RBD of SARS-CoV-2 at resolutions of 2.55 and 2.70 Å, allowing detailed characterization of the target epitopes and the SARS-CoV-2 neutralization mechanism of these two mAbs. SARS-CoV-2 mAbs can compete with ACE2 binding and exert neutralizing activity by inhibiting virus particle binding to host cells (Barnes et al., 2020; Brouwer et al., 2020; Cao et al., 2020; Ju et al., 2020; Kreer et al., 2020; Robbiani et al., 2020; Rogers et al., 2020; Wec et al., 2020), a key mechanism identified previously in SARS-CoV-neutralizing antibodies (Prabakaran et al., 2006; ter Meulen et al., 2006). Steric hindrance of mAbs blocking ACE2 binding to the RBD provides one mechanistic explanation of virus neutralization (Barnes et al., 2020; Cao et al., 2020; Wu et al., 2020). CV07-250 clearly belongs to this category of antibodies because its epitope lies within the ACE2 binding site, and it approaches the RBD from a similar angle as ACE2. In contrast, the epitope of CV07-270 only partially overlaps with the ACE2 binding site and approaches the RBD ridge from a different angle. In line with these findings, competition of CV07-270 with ACE2 binding, as detected by ELISA, was very weak; therefore, its mechanism of virus neutralization remains elusive. Of note, there have been reports of neutralizing antibodies targeting epitopes distant to the ACE2 binding site (Chi et al., 2020). Future research will need to clarify whether additional mechanisms, like triggering conformational changes in the spike protein upon antibody binding, contribute to virus neutralization, as reported for SARS-CoV (Walls et al., 2019).

Second, the majority of our SARS-CoV-2 mAbs are close to germline configuration, supporting previous studies (Kreer et al., 2020; Robbiani et al., 2020). Binding of some antibodies to HEp-2 cells has been reported before (Kreer et al., 2020), a finding we could confirm in our cohort. Given the increased probability of autoreactivity of near-germline antibodies, we additionally investigated reactivity of SARS-CoV-2 mAbs with unfixed murine tissue, allowing detection of reactivity to potential self-antigens in their natural conformation. Indeed, we found that a fraction of SARS-CoV-2-neutralizing antibodies also bound to brain-, lung-, heart-, kidney-, or gut-expressed epitopes. Such reactivity with host antigens should ideally be prevented by immunological tolerance mechanisms, but complete exclusion of such antibodies would generate “holes” in the antibody repertoire. In fact, HIV utilizes epitopes shared by its envelope and mammalian self-antigens, harnessing immunological tolerance to impair anti-HIV antibody responses (Yang et al., 2013) and impeding successful vaccination (Jardine et al., 2016). To defy virus escape in HIV and, similarly, COVID-19, anergic, strongly self-reactive B cells likely enter germinal centers and undergo clonal redemption to mutate away from self-reactivity while retaining HIV or SARS-CoV-2 binding (Reed et al., 2016). Interestingly, longitudinal analysis of mAbs in COVID-19 showed that the number of SHMs in SARS-CoV-2-neutralizing antibodies only increased marginally over time (Kreer et al., 2020). This finding suggests that the self-reactivity observed in this study may not be limited to mAbs of the early humoral immune response in SARS-CoV-2 infection. Whether self-reactive antibodies could contribute to extra-pulmonary symptoms in COVID-19 awaits further studies and should be closely monitored in vaccination trials.

Finally, we evaluated in detail the *in vivo* efficacy of the most potent neutralizing antibody, CV07-209, in a Syrian hamster model of SARS-CoV-2 infection. This model is characterized by a severe phenotype including weight loss and distinct lung pathology. Our results demonstrated that prophylaxis and treatment with a single dose of CV07-209 not only led to clinical improvement, as shown by the absence of weight loss, but also to markedly reduced lung pathology. Although the findings confirm the efficacy of prophylactic mAb administration as described by other groups in mice, hamsters, and rhesus macaques (Cao et al., 2020; Liu et al., 2020; Rogers et al., 2020; Zost et al., 2020), our work also demonstrates the efficacy of post-exposure treatment in hamsters leading to virus clearance, clinical remission, and prevention of lung injury. We provide detailed insights into the lung pathology of SARS-CoV-2-infected hamsters at multiple times during the disease course, including the regeneration phase. It complements two very recent demonstrations of a therapeutic effect of mAbs in a hamster model of COVID-19 (Baum et al., 2020; Li et al., 2020). These data expand the growing knowledge about post-exposure treatment from transgenic hACE2 mice (Cao et al., 2020) and a mouse model using adenovector delivery of human ACE2 before virus challenge (Liu et al., 2020). Collectively, our results indicate that mAb treatment can be fine-tuned for exclusion of self-reactivity with mammalian tissues and that mAb administration can also be efficacious after infection, which will be the prevailing setting in COVID-19 patients.

### Limitations of Study

Although our study confirms the potential of therapeutic mAb application for treatment of COVID-19, interpretation of the data is limited to a first exploration of a short window between infection and antibody administration. Although our paradigm mimics the relevant scenario of immediate post-exposure treatment, we cannot conclude whether the therapeutic benefit can also be translated into the more common clinical setting of treatment at heterogenous time points after symptoms have occurred. For this, follow-up studies will have to focus on delayed mAb application after symptom onset.

We also describe the reactivity of SARS-CoV-2 mAbs to self-antigens from different tissues. These findings require attention and, simultaneously, careful interpretation and thorough investigation to provide a better understanding of their functional relevance beyond the observed binding. This includes identification of non-viral target antigens, functional *in vitro* studies, and *in vivo* models. The self-reactive mAbs identified in this study derived from patients without severe extra-pulmonary symptoms. To address a possible connection between self-reactive antibodies and the diverse clinical manifestations of COVID-19, expression and characterization of mAbs from patients with such disease courses are needed.

## STAR★Methods

### Key Resources Table

**Table:** 

REAGENT or RESOURCE	SOURCE	IDENTIFIER
**Antibodies**		

mouse anti-CD19-BV786 (clone SJ25C1)	BD Biosciences	Cat# 563326; RRID: AB_2744314
mouse anti-CD27-PE (clone M-T271)	BD Biosciences	Cat# 555441; RRID: AB_395834
mouse anti-CD38-FITC (clone HIT2)	BD Biosciences	Cat# 560982; RRID: AB_2033957
mouse anti-CD19-PE (clone HIB19)	Biolegend	Cat# 302207; RRID: AB_314237
mouse anti-CD38-PEcy7 (clone HIT2)	Biolegend	Cat# 303505; RRID: AB_314357
mouse anti-CD27-APC (clone O323)	Biolegend	Cat# 302809; RRID: AB_314301
donkey anti-rabbit IgG	Dianova	Cat# 711-005-152; RRID: AB_2340585
donkey anti-human IgG-HRPO	Dianova	Cat# 709-035-149; RRID: AB_2340495
donkey F(ab’)2 anti-rabbit IgG-HRPO	Dianova	Cat# 711-036-152; RRID: AB_2340590
mouse anti-HA.11 (clone 16B12)	Biolegend	Cat# 901515; RRID: AB_2565334
donkey anti-mouse IgG-HRPO	Dianova	Cat# 715-035-150; RRID: AB_2340770
mouse anti-human IgG (clone MT145)	Mabtech	Cat# 3850-1-250; RRID: AB_10697677
mouse anti-human IgG-ALP (clone MT78)	Mabtech	Cat# 3850-9A; RRID: AB_10697678
goat-anti human IgG-Alexa Fluor 488	Dianova	Cat# 109-545-088; RRID: AB_2337838
goat anti-human IgG-Alexa Fluor 488	Dianova	Cat# 109-545-003; RRID: AB_2337831
mouse anti-Smooth Muscle Actin (clone 1A4)	Agilent	Cat# M085129-2; RRID: AB_2811108
goat anti-mouse IgG-Alexa Fluor 594	Dianova	Cat# 115-585-003; RRID: AB_2338871

**Bacterial and Virus Strains**		

10-beta Competent *E. coli* (High Efficiency)	New England Biolabs	Cat#C3019I
SARS-CoV-2	Wölfel et al., 2020	BetaCoV/Germany/BavPat1/2020

**Biological Samples**		

PBMCs and serum, donor CV01	Kurth et al., 2020	N/A
PBMCs and serum, donor CV03	Kurth et al., 2020	N/A
PBMCs and serum, donor CV05	Kurth et al., 2020	N/A
PBMCs and serum, donor CV07	Kurth et al., 2020	N/A
PBMCs and serum, donor CV23	Kurth et al., 2020	N/A
PBMCs and serum, donor CV24	Kurth et al., 2020	N/A
PBMCs and serum, donor CV38	Kurth et al., 2020	N/A
PBMCs and serum, donor CV48	Kurth et al., 2020	N/A
PBMCs and serum, donor CVX1	This paper	N/A
PBMCs and serum, donor CVX2	This paper	N/A
Hamster lungs, swabs, nasal washes	This paper	N/A

**Chemicals, Peptides, and Recombinant Proteins**		

CruzFluor 647 succinimidyl ester	Santa Cruz Biotechnology	Cat#sc-362620
7-Aminoactinomycin D (7-AAD)	Thermo Fisher	Cat#A1310
RNasin Plus RNase Inhibitor	Promega	Cat#N2615
dNTP Set	Thermo Fisher	Cat#R1121
DTT, 1M	Thermo Fisher	Cat#P2325
Phosphate-buffered saline (PBS), 10X	GIBCO	Cat#70011051
SuperScript II Reverse Transcriptase-4	Life Technologies	Cat#18064071
HotStarTaq DNA Polymerase	QIAGEN	Cat#203209
Q5 Hot Start High-Fidelity DNA Polymerase	New England Biolabs	Cat#M0493L
Agarose Broad Range ROTIagarose	Carl Roth	Cat#846.3
Terrific Broth Powder (TB Medium)	PanReac AppliChem	Cat#A0974
LB Broth, granulated,for molecular biology	Carl Roth	Cat#6673.4
Ampicillin Sodium Salt > 97%	Carl Roth	Cat#K029.1
Aqua Ad Injectabilia	B. Braun	Cat#235 1744
DMEM, high glucose, GlutaMAX Supplement, pyruvate	Life Technologies	Cat#31966-047
MEM non-essential amino acid solution (100x)	Sigma Aldrich	Cat#M7145
Fetal Bovine Serum	Sigma Aldrich	Cat#F7524
0,05% Trypsin EDTA phenol red	Life Technologies	Cat#25300-054
Penicillin-Streptomycin	Sigma Aldrich	Cat#P0781
Polyethylenimine, branched	Sigma Aldrich	Cat#408727
Protein G Sepharose 4 Fast flow	GE Healthcare	Cat#17-0618-01
Albumin Fraktion V Powder, protease free	Carl Roth	Cat#T844.2
Bovine Serum Albumin (IgG-free, Protease-free)	Dianova	Cat#001-000-161
StartingBlock (PBS) Blocking Buffer	Thermo Fisher	Cat#37538
Tween 20	Applichem	Cat#A4974
1-step Ultra TMB-ELISA	Thermo Fisher	Cat#34028
1-step Slow TMB-ELISA	Thermo Fisher	Cat#34024
Streptavidin-POD conjugate	Roche Diagnostics	Cat#11089153001
Fugene HD	Roche	Cat#E231A
SARS-CoV-2 S protein-RBD-mFC	Acrobiosystems	Cat# SPD-C5259
SARS-CoV-2-S1	Creative Diagnostics	Cat#DAGC091
DpnI	New England Biolabs	Cat#R0176L
Sodium chloride (NaCl)	Sigma-Aldrich	Cat#S9888
Tris Base	Sigma-Aldrich	Cat#11814273001
Concentrated hydrochloric acid (HCl)	Sigma-Aldrich	Cat#H1758
Opti-MEM I reduced serum media	GIBCO	Cat#51985091
Phosphate-buffered saline (PBS)	Thermo Fisher	Cat#14040133
Ni-NTA Superflow	QIAGEN	Cat#30450
Bovine Serum Albumin (BSA)	Sigma-Aldrich	Cat#A9418
Tween 20	Fisher Scientific	Cat#BP337-500
Chemicals for protein crystallization	Hampton Research	Cat#N/A
CaptureSelect CH1-XL Affinity Matrix	Thermo Fisher	Cat#2943452010
Normal Goat Serum	Abcam	Cat#ab138478
DRAQ5	Abcam	Cat#ab108410
Paraformaldehyd	Alfa Aesar	Cat#J61899
Roti®-Mount Fluor-Care DAPI	Carl Roth	Cat#HP20.1

**Critical Commercial Assays**		

BD Vacutainer® CPT Mononuclear Cell Preparation Tube	Becton Dickinson	Cat#362782
NEBuilder HiFi DNA Assembly Master Mix	New England Biolabs	Cat#E2621X
NucleoBond Xtra Maxi kit for transfection-grade plasmid DNA	Macherey-Nagel	Cat#740414
NucleoSpin 96 Plasmid Kit for plasmid DNA purification Transfection grade	Macherey-Nagel	Cat#740491.4
Human IgG ELISA development kit (ALP)	Mabtech	Cat#3850-1AD-6
In-Fusion HD Cloning Kit	Takara	Cat#639647
PCR Clean-Up and Gel Extraction Kit	Clontech Laboratories	Cat#740609.250
QIAprep Spin Miniprep Kit	QIAGEN	Cat#27106
Anti-SARS-CoV-2 ELISA IgG	EUROIMMUN	Cat#EI 2606-9601 G
EZ-Link Sulfo-NHS-SS-Biotin- und Markierungs-Kits	Thermo Fisher	Cat#21331
ExpiCHO Expression System Kit	Thermo Fisher	Cat#A29133
Nova-Lite HEp-2 ANA Kit	Werfen	Cat#066708101
Pierce 3K Protein Concentrator PES	Thermo Fisher	Cat#88525
innuPREP Virus RNA Kit	Analytic Jena	Cat#845-KS-4700250
NEB Luna Universal Probe One-Step RT-qPCR Kit	New England Biolabs	Cat#E3006L
Platinum SuperScript III RT-PCR-System	Thermo Fisher	Cat#12574018
ViewRNA ISH Tissue Assay Kit	Thermo Fisher	Cat#19931

**Deposited Data**		

X-ray coordinates and structure factors of CV07-250/RBD complex	This paper	PDB: 6XKQ
X-ray coordinates and structure factors of CV07-270/RBD complex	This paper	PDB: 6XKP
Nucleotide sequences of top 18 antibodies	This paper	GenBank:MW002770 - MW002805
Raw sequencing and analysis data from all isolated antibodies	This paper	https://doi.org/10.24433/CO.1724316.v1
Tables S1, S2, and S3–S6	This paper	https://doi.org/10.17632/f6tb3csgjt.1

**Experimental Models: Cell Lines**		

HEK293 cells	DSMZ	Cat#ACC 305
VeroE6 cells	ATCC	Cat#CRL-1586
VeroB4 cells	DSMZ	Cat#ACC 33
ExpiCHO cells	Thermo Fisher	Cat#A29127
Sf9 cells	ATCC	Cat#CRL-1711
High Five cells	Thermo Fisher	Cat#B85502

**Experimental Models: Organisms/Strains**		

Syrian hamster, *Mesocricetus auratus*	Janvier	RjHan:Aura

**Oligonucleotides**		

random hexamer primer p(dN)6	Roche	Cat#11034731001
PCR Primer	Kreye et al., 2016	N/A
gH5 2/3-1 (ATCCTTTTTCTAGTAGCAACTGCAAC CGGTGTACATTCCCAGGTGCAGCTGGTGCAG)	This paper	N/A
gH5 2/3-1 (ATCCTTTTTCTAGTAGCAACTGCAAC CGGTGTACATTCCCAGGTGCAGCTGGTGCAG)	This paper	N/A
gH5 3-7 (ATCCTTTTTCTAGTAGCAACTGCAAC CGGTGTACATTCCCAGGTTCAGCTGGTGCAG)	This paper	N/A
gH5 3-8 (ATCCTTTTTCTAGTAGCAACTGCAAC CGGTGTACATTCCCAGGTCCAGCTGGTACAG)	This paper	N/A
gH5 2/3-2 (ATCCTTTTTCTAGTAGCAACTGCAAC CGGTGTACATTCCGAGGTGCAGCTGGTGCAG)	This paper	N/A
gH5 2/3-3 (ATCCTTTTTCTAGTAGCAACTGCAAC CGGTGTACATTCTGAGGTGCAGCTGGTGGAG)	This paper	N/A
gH5 3-9 (ATCCTTTTTCTAGTAGCAACTGCAAC CGGTGTACATTCTGAAGTGCAGCTGGTGGAG)	This paper	N/A
gH5 2/3-4 (ATCCTTTTTCTAGTAGCAACTGCAAC CGGTGTACATTCTGAGGTGCAGCTGTTGGAG)	This paper	N/A
gH5 3-10 (ATCCTTTTTCTAGTAGCAACTGCAAC CGGTGTACATTCTCAGGTGCAGCTGGTGGAG)	This paper	N/A
gH5 2/3-5 (ATCCTTTTTCTAGTAGCAACTGCAAC CGGTGTACATTCCCAGGTGCAGCTGCAGGAG)	This paper	N/A
gH5 2/3-6 (ATCCTTTTTCTAGTAGCAACTGCAAC CGGTGTACATTCCCAGGTGCAGCTACAGCAGTG)	This paper	N/A
gH5 3-11 (ATCCTTTTTCTAGTAGCAACTGCAAC CGGTGTACATTCCCAGCTGCAGCTGCAGGAG)	This paper	N/A
gH5 3-12 (ATCCTTTTTCTAGTAGCAACTGCAAC CGGTGTACATTCCCAGGTACAGCTGCAGCAG)	This paper	N/A
gH5 3-13 (ATCCTTTTTCTAGTAGCAACTGCAAC CGGTGTACATTCTCAGGTGCAGCTGGTGCAATCTGG)	This paper	N/A
gH5 3-14 (ATCCTTTTTCTAGTAGCAACTGCAAC CGGTGTACATTCCGAAGTGCAGCTGGTGCAG)	This paper	N/A
gH5 3-15 (ATCCTTTTTCTAGTAGCAACTGCAAC CGGTGTACATTCCCAGGTCCAGCTTGTGCAG)	This paper	N/A
gH5 3-16 (ATCCTTTTTCTAGTAGCAACTGCAAC CGGTGTACATTCCCAGGTCCAGCTGGTGCAG)	This paper	N/A
gH3 3-1 (GGAAGACCGATGGGCCCTTGGTCGAC GCTGAGGAGACGGTGACCAG)	This paper	N/A
gH3 3-2 (GGAAGACCGATGGGCCCTTGGTCGAC GCTGAAGAGACGGTGACCATTG)	This paper	N/A
gH3 3-3 (GGAAGACCGATGGGCCCTTGGTCGAC GCTGAGGAGACGGTGACCGTG)	This paper	N/A
gK5 3-1 (ATCCTTTTTCTAGTAGCAACTGCAAC CGGTGTACATTCTGACATCCAGATGACCCAGTC)	This paper	N/A
gK5 3-2 (ATCCTTTTTCTAGTAGCAACTGCAACC GGTGTACATTCAGACATCCAGTTGACCCAGTCT)	This paper	N/A
gK5 3-3 (ATCCTTTTTCTAGTAGCAACTGCAAC CGGTGTACATTGTGCCATCCGGATGACCCAGTC)	This paper	N/A
gK5 3-4 (ATCCTTTTTCTAGTAGCAACTGCAAC CGGTGTACATGGGGATATTGTGATGACCCAGAC)	This paper	N/A
gK5 3-5 (ATCCTTTTTCTAGTAGCAACTGCAAC CGGTGTACATGGGGATATTGTGATGACTCAGTC)	This paper	N/A
gK5 3-6 (ATCCTTTTTCTAGTAGCAACTGCAAC CGGTGTACATGGGGATGTTGTGATGACTCAGTC)	This paper	N/A
gK5 3-7 (ATCCTTTTTCTAGTAGCAACTGCAAC CGGTGTACATTCAGAAATTGTGTTGACACAGTC)	This paper	N/A
gK5 3-8 (ATCCTTTTTCTAGTAGCAACTGCAAC CGGTGTACATTCAGAAATAGTGATGACGCAGTC)	This paper	N/A
gK5 3-9 (ATCCTTTTTCTAGTAGCAACTGCAAC CGGTGTACATTCAGAAATTGTGTTGACGCAGTCT)	This paper	N/A
gK5 3-10 (ATCCTTTTTCTAGTAGCAACTGCAAC CGGTGTACATTCGGACATCGTGATGACCCAGTC)	This paper	N/A
gK5 3-11 (ATCCTTTTTCTAGTAGCAACTGCAAC CGGTGTACATTGTGTCATCTGGATGACCCAGTC)	This paper	N/A
gK5 3-12 (ATCCTTTTTCTAGTAGCAACTGCAAC CGGTGTACATTCTGCCATCCAGTTGACCCAGTC)	This paper	N/A
gK3 3-1 (AAGACAGATGGTGCAGCCACCGTACG TTTGATYTCCACCTTGGTC)	This paper	N/A
gK3 3-2 (AAGACAGATGGTGCAGCCACCGTACG TTTGATCTCCAGCTTGGTC)	This paper	N/A
gK3 3-3 (AAGACAGATGGTGCAGCCACCGTACG TTTGATATCCACTTTGGTC)	This paper	N/A
gK3 3-4 (AAGACAGATGGTGCAGCCACCGTACG TTTAATCTCCAGTCGTGTC)	This paper	N/A
gL5 2/3-1 (ATCCTTTTTCTAGTAGCAA CTGCAAC CGGTTCCTGGGCCCAGTCTGTGCTGACKCAG)	This paper	N/A
gL5 2/3-2 (ATCCTTTTTCTAGTAGCAA CTGCAAC CGGTTCCTGGGCCCAGTCTGCCCTGACTCAG)	This paper	N/A
gL5 2/3-3 (ATCCTTTTTCTAGTAGCAA CTGCAAC CGGTTCTGTGACCTCCTATGAGCTGACWCAG)	This paper	N/A
gL5 2/3-4 (ATCCTTTTTCTAGTAGCAA CTGCAAC CGGTTCTCTCTCSCAGCYTGTGCTGACTCA)	This paper	N/A
gL5 2/3-5 (ATCCTTTTTCTAGTAGCAA CTGCAAC CGGTTCTTGGGCCAATTTTATGCTGACTCAG)	This paper	N/A
gL5 2/3-6 (ATCCTTTTTCTAGTAGCAA CTGCAAC CGGTTCCAATTCYCAGRCTGTGGTGACYCAG)	This paper	N/A
gL3 2/3 (TGTTGGCTTGAAGCTCCTCACTCGAGG GYGGGAACAGAGTG)	This paper	N/A
E_Sarbeco_F (ACAGGTACGTTAATAGTTAATAGCGT)	Corman et al., 2020	N/A
E_Sarbeco_R (5′-ATATTGCAGCAGTACGCACACA)	Corman et al., 2020	N/A
E_Sarbeco_P (FAM-ACACTAGCCATCCTTACTGCGCT TCG-BBQ)	Corman et al., 2020	N/A
RPL18_F (GTTTATGAGTCGCACTAACCG)	Zivcec et al., 2011	N/A
RPL18_R (TGTTCTCTCGGCCAGGAA)	Zivcec et al., 2011	N/A
RPL18_P (YAK-TCTGTCCCTGTCCCGGATGATC-BBQ)	Zivcec et al., 2011	N/A
SARS-CoV-2_sgRNA_F; (CGATCTCTTGTAGATCTG TTCTC)	Wölfel et al., 2020	N/A
ATATTGCAGCAGTACGCACACA-	Wölfel et al., 2020	N/A
FAM-ACACTAGCCATCCTTACTGCGCTTCG-BBQ	Wölfel et al., 2020	N/A
Synthetic probes for the *in situ*-detection of the N gene RNA of SARS-CoV-2	Thermo Fisher	Cat#VPNKRHM

**Recombinant DNA**		

Human antibody expression vectors (IgG1, Igλ, Igκ)	Tiller et al., 2008	N/A
Plasmid for expression of rabbit Fc-tagged GluN1-ATD	Kornau et al., 2020	N/A
Plasmid for expression of rabbit Fc-tagged SARS-CoV-2 RBD	This paper	N/A
Plasmid for expression of rabbit Fc-tagged SARS-CoV RBD	This paper	N/A
Plasmid for expression of rabbit Fc-tagged MERS-CoV RBD	This paper	N/A
Plasmid for expression of rabbit Fc-tagged HCoV-229E RBD	This paper	N/A
Plasmid for expression of rabbit Fc-tagged HCoV-NL63 RBD	This paper	N/A
Plasmid for expression of rabbit Fc-tagged HCoV-HKU1 RBD	This paper	N/A
Plasmid for expression of rabbit Fc-tagged HCoV-OC43 RBD	This paper	N/A
Plasmid for expression of His- and HA-tagged extracellular domain of human ACE2	This paper	N/A
pCG1-SARS-CoV-2-S	Hoffmann et al., 2020	N/A
pCG1-MERS-CoV-S	Buchholz et al., 2013	N/A
pCG1-HCoV-229E-S	Buchholz et al., 2013	N/A
pCG1-HCoV-NL63-S	Buchholz et al., 2013	N/A
pCG1-HCoV-OC43-S	Buchholz et al., 2013	N/A
pCG1-HCoV-HKU1-S	Buchholz et al., 2013	N/A

**Software and Algorithms**		

FACSDiva Software Version 8.0.2	BD Bioscience	Cat#659523
BASE Software	Reincke et al., 2020	https://github.com/automatedSequencing/BASE
Skanit Software 3.2	Thermo Fisher	Cat#5187139
Biacore T200 Version 3.2	Cytiva	http://www.cytivalifesciences.com/country-selection?originalItemPath=%2f
HKL2000	Otwinowski and Minor, 1997	N/A
Phaser	McCoy et al., 2007	N/A
Coot	Emsley and Cowtan, 2004	N/A
Phenix	Adams et al., 2010	N/A
PISA	Krissinel and Henrick, 2007	N/A
Microsoft Excel, Word, PowerPoint	Microsoft Office	https://www.microsoft.com/en-us/
GraphPad Prism, Version 8.4	GraphPad Software	https://www.graphpad.com
Biorender	BioRender	https://biorender.com
PDBePISA	Europea Bioinformatics Institute	https://www.ebi.ac.uk/pdbe/prot_int/pistart.html
CellSens Imaging Software, Version 1.18	Olympus	https://www.olympus-lifescience.com/en/

**Other**		

BD FACSAria II SORP	BD Bioscience	https://www.bdbiosciences.com/en-us

### Resource Availability

#### Lead Contact

Further information and requests for resources and reagents should be directed to and will be fulfilled by the Lead Contact, Jakob Kreye (jakob.kreye@dzne.de).

#### Materials Availability

All requests for materials including antibodies, viruses, plasmids and proteins generated in this study should be directed to the Lead Contact author. Materials will be made available under a Material Transfer Agreement (MTA) for non-commercial usage.

#### Data and Code Availability

X-ray coordinates and structure factors are deposited at the RCSB Protein Data Bank under accession codes PDB: 6XKQ and PDB: 6XKP. The accession number for the nucleotide sequences of the top 18 antibodies reported in this paper is GenBank: MW002770 - MW002805The. The raw sequencing data associated with this manuscript together with the analysis using custom BASE software have been deposited to Code Ocean (https://codeocean.com/capsule/7823731/tree/v1, https://doi.org/10.24433/CO.1724316.v1). The software used for Ig sequence analysis is available at https://github.com/automatedSequencing/BASE. The published article includes all data generated or analyzed during this study and are available from the corresponding author on request.

### Experimental Models and Subject Details

#### SARS-CoV-2-infected individuals and sample collection

The patients have given written informed consent and analyses were approved by the Institutional Review Board of Charité - Universitätsmedizin Berlin, corporate member of Freie Universität Berlin, Humboldt-Universität Berlin, and Berlin Institute of Health, Berlin. All patients in this study were tested positive for SARS-CoV-2 infection by RT-PCR. Most patients belong to a prospective COVID-19 cohort (Kurth et al., 2020). Patient characteristics are described in Table S1.

#### Animal experiment approval and animal care

The animal experiment was approved by the Landesamt für Gesundheit und Soziales in Berlin, Germany (approval number 0086/20) and performed in compliance with relevant national and international guidelines for care and humane use of animals. *In vitro* and animal work was conducted under appropriate biosafety precautions in a BSL-3 facility at the Institute of Virology, Freie Universität Berlin, Germany. Twenty-seven six-week old female and male golden Syrian hamsters (Mesocricetus auratus; outbred hamster strain RjHan:AURA, Janvier Labs) were kept in groups of 3 animals in enriched, individually ventilated cages. The animals had *ad libitum* access to food and water and were allowed to acclimate to these conditions for seven days prior to prophylactic treatment and infection. Cage temperatures and relative humidity were recorded daily and ranged from 22-24°C and 40%–55%, respectively.

### Method Details

#### PBMC collection and FACS staining

Recombinant SARS-CoV-2-S1 protein produced in HEK cells (Creative Diagnostics, DAGC091) was covalently labeled using CruzFluor647 (Santa Cruz Biotechnology, sc-362620) according to the manufacturer’s instructions.

Using fluorescence-activated cell sorting we sorted viable single cells from freshly isolated peripheral blood mononuclear cells (PBMCs as 7AAD^-^CD19^+^CD27^+^CD38^+^ antibody-secreting cells (ASCs) or SARS-CoV2-S1-enriched 7AAD^-^CD19^+^CD27^+^ memory B cells (MBCs) into 96-well PCR plates. Staining was performed on ice for 25 minutes in PBS with 2% FCS using the following antibodies: 7-AAD 1:400 (Thermo Fisher Scientific), CD19-BV786 1:20 (clone SJ25C1, BD Biosciences, 563326), CD27-PE 1:5 (clone M-T271, BD Biosciences, 555441), CD38-FITC 1:5 (clone HIT2, BD Biosciences, 560982), and SARS-CoV-S1-CF647 at 1 μg/ml for patients CV07, CV38, CV23, CV24, CV 38, CV48, CV-X1, CV-X2 and CV01 (second time point, fig. S1). The first patients (CV01 (first time point), CV03, and CV05) were stained with a divergent set of antibodies: CD19-PE 1:50 (clone HIB19, BioLegend, 302207), CD38-PEcy7 1:50 (clone HIT2, BioLegend, 303505), CD27-APC 1:50 (clone O323, BioLegend, 302809) and DAPI as viability dye.

#### Generation of recombinant human monoclonal antibodies

Monoclonal antibodies were generated following established protocols (Kornau et al., 2020; Kreye et al., 2016; Tiller et al., 2008) with modifications as mentioned. We used a nested PCR strategy to amplify variable domains of immunoglobulin heavy and light chain genes from single cell cDNA and analyzed sequences with aBASE module of customized Brain Antibody Sequence Evaluation (BASE) software (Reincke et al., 2020). Pairs of functional Ig genes were PCR-amplified using specific primers with Q5 Polymerase (NEB). PCR-product and linearized vector were assembled using Gibson cloning with HiFi DNA Assembly Master Mix (NEB). Cloning was considered successful when sequence identify > 99.5% was given, verified by the cBASE module of BASE software. For mAb expression, human embryonic kidney cells (HEK293T) were transiently transfected with matching Ig heavy and light chains. Three days later mAb containing cell culture supernatant was harvested. Ig concentrations were determined and used for reactivity and neutralization screening, if Ig concentration was above 1 μg/ml. For biophysical characterization assays and *in vivo* experiments, supernatants were purified using Protein G Sepharose beads (GE Healthcare), dialyzed against PBS and sterile-filtered using 0.2 μm filter units (GE Healthcare). For *in vivo* experiments, mAbs were concentrated using Pierce 3K Protein Concentrator PES (Thermo Scientific).

#### SARS-CoV-2-S1 ELISA

Screening for SARS-CoV-2-specific mAbs was done by using anti-SARS-CoV-2-S1 IgG ELISAs (EUROIMMUN Medizinische Labordiagnostika AG) according to the manufacturer’s protocol. mAb containing cell culture supernatants were pre-diluted 1:5, patient sera 1:100. Optical density (OD) ratios were calculated by dividing the OD at 450 nm by the OD of the calibrator included in the kit. OD ratios of 0.5 were considered reactive.

#### RBD ELISA

Binding to the receptor-binding domain (RBD) of S1 was tested in an ELISA. To this end, a fusion protein (RBD-Fc) of the signal peptide of the NMDA receptor subunit GluN1, the RBD-SD1 part of SARS-CoV2-S1 (amino acids 319-591) and the constant region of rabbit IgG1 heavy chain (Fc) was expressed in HEK293T cells and immobilized onto 96-well plates from cell culture supernatant via anti-rabbit IgG (Dianova, 711-005-152) antibodies. Then, human mAbs were applied and detected using horseradish peroxidase (HRP)-conjugated anti-human IgG (Dianova, 709-035-149) and the HRP substrate 1-step Ultra TMB (Thermo Fisher Scientific, Waltham, MA). All S1+ mAbs were screened at a human IgG concentration of 10 ng/ml to detect strong RBD binders and the ones negative at this concentration were re-evaluated for RBD reactivity using a 1:5 dilution of the cell culture supernatants. To test for specificity within the coronavirus family, we expressed and immobilized Fc fusion proteins of the RBD-SD1 regions of SARS-CoV, MERS-CoV and the endemic human coronaviruses HCoV-229E, HCoV-NL63, HCoV-HKU1, and HCoV-OC43 and applied mAbs at 1 μg/ml. The presence of immobilized antigens was confirmed by incubation with HRP-conjugated anti-rabbit IgG (Dianova, 711-036-152). Assays for concentration-dependent RBD binding (Figure 1E) were developed using 1-step Slow TMB (Thermo Fisher Scientific). EC_50_ was determined from non-linear regression models using Graph Pad Prism 8.

To evaluate the ability of mAbs to interfere with the binding of ACE2 to SARS-CoV-2 RBD, we expressed ACE2-HA, a fusion protein of the extracellular region of human ACE2 (amino acids 1-615) followed by a His-tag and a hemagglutinin (HA)-tag in HEK293T cells and applied it in a modified RBD ELISA. Captured RBD-Fc was incubated with mAbs at 0.5 μg/ml for 15 minutes and subsequently with ACE2-HA-containing cell culture supernatant for 1 h. ACE2-HA binding was detected using anti-HA antibody HA.11 (clone 16B12, BioLegend, San Diego, CA, 901515), HRP-conjugated anti-mouse IgG (Dianova, 715-035-150) and 1-step UltraTMB.

For experiments regarding the competition between mAbs for RBD binding, purified monoclonal antibodies were biotinylated using EZ-Link Sulfo-NHS-Biotin (Thermo Fisher) according to the manufacturer’s instructions. Briefly, 50-200 μg of purified antibody were incubated with 200-fold molar excess Sulfo-NHS-Biotin for 30 minutes at room temperature. Excess Sulfo-NHS-Biotin was removed by dialysis for 16 hours. Recovery rate of IgG ranged from 60%–100%. RBD-Fc captured on ELISA plates was incubated with mAbs at 10 μg/ml for 15 minutes. Then, one volume of biotinylated mcAbs at 100 ng/ml was added and the mixture incubated for additional 15 minutes, followed by detection using HRP-conjugated streptavidin (Roche Diagnostics) and 1-step Ultra TMB. Background by the HRP-conjugated detection antibodies alone was subtracted from all absorbance values.

#### Circos plot of public clonotypes

Antibodies which share same V and J gene on both Ig heavy and light chain are considered to be one clonotype. Such clonotypes are considered *public* if they are identified in different patients. After identification of public clonotypes, they were plotted in a Circos plot using the R package circlize (Gu et al., 2014).

#### Identification of 18 strongly neutralizing antibodies

To identify the most potent SARS-CoV-2 neutralizing mAb, all 122 S1-reactive mAbs were screened for binding to RBD. 87 were defined as strongly binding to RBD (defined as detectable binding at 10 ng/ml in an RBD ELISA) and then assessed for neutralization of authentic SARS-CoV-2 at 25 and 250 ng/ml using mAb-containing cell culture supernatants. Antibodies were further selected (i) as the strongest neutralizing mAb of the respective donor and / or (ii) with an estimated IC_50_ of 25 ng/ml or below and / or (iii) with an estimated IC_90_ of 250 ng/ml or below. These were defined as the 18 most potent antibodies (top 18) and expressed as purified antibodies for detailed biophysical characterization.

#### Surface plasmon resonance measurements

The antigen (SARS-CoV-2 S protein-RBD-mFc, Accrobiosystems) was reversibly immobilized on a C1 sensor chip via anti-mouse IgG. Purified mAbs were injected at different concentrations in a buffer consisting of 10 mM HEPES pH 7.4, 150 mM NaCl, 3 mM EDTA, 0.05% Tween 20. CV-X1-126 and CV38-139 were analyzed in a buffer containing 400 mM NaCl as there was a slight upward drift at the beginning of the dissociation phase due to non-specific binding of to the reference flow. Multi-cycle-kinetics analyses were performed in duplicates except for non-neutralizing CV03-191. K_a_, K_d_ and K_D_-values were determined using a monovalent analyte model. Recordings were performed on a Biacore T200 instrument at 25°C.

#### Plaque reduction neutralization test

To detect neutralizing activity of SARS-CoV-2-specific mAbs, plaque reduction neutralization tests (PRNT) were done as described before (Wölfel et al., 2020). Briefly, Vero E6 cells (1.6 x10^5^ cells/well) were seeded in 24-well plates and incubated overnight. For each dilution step, mAbs were diluted in OptiPro and mixed 1:1 with 200 μL virus (Munich isolate 984) (Wölfel et al., 2020) solution containing 100 plaque forming units. The 400 μL mAb-virus solution was vortexed gently and incubated at 37°C for 1 hour. Each 24-well was incubated with 200 μL mAb-virus solution. After 1 hour at 37°, the supernatants were discarded and cells were washed once with PBS and supplemented with 1.2% Avicel solution in DMEM. After 3 days at 37°C, the supernatants were removed and the 24-well plates were fixed and inactivated using a 6% formaldehyde/PBS solution and stained with crystal violet. All dilutions were tested in duplicates. For PRNT-screening mAb dilutions of 25 and 250 ng of IgG/ml were assessed. IC_50_ was determined from non-linear regression models using Graph Pad Prism 8.

#### Immunocytochemistry

Recombinant spike protein-based immunofluorescence assays were done as previously described (Buchholz et al., 2013; Corman et al., 2020; Wölfel et al., 2020). Briefly, VeroB4 cells were transfected with previously described pCG1 plasmids encoding SARS-CoV-2, MERS-CoV, HCoV-NL63, −229E, -OC43, and -HKU1 spike proteins (Buchholz et al., 2013; Hoffmann et al., 2020). For transfection, Fugene HD (Roche) was used in a Fugene to DNA ratio of 3:1. After 24 hours, transfected as well as untransfected VeroB4 cells were harvested and resuspended in DMEM/10% FCS to achieve a cell density of 2.5x10^5^ cells/ml each. Transfected and untransfected VeroB4 cells were mixed 1:1 and 50 μL of the cell suspension was applied to each incubation field of a multitest cover slide (Dunn Labortechnik). The multitest cover slides were incubated for 6 hours before they were washed with PBS and fixed with ice-cold acetone/methanol (ratio 1:1) for 10 minutes. For the immunofluorescence test, the incubation fields were blocked with 5% non-fat dry milk in PBS/0.2% Tween for 60 minutes. Purified mAbs were diluted in EUROIMMUN sample buffer to a concentration of 5 μg/ml and 30 μL of the dilution was applied per incubation field. After 1 hour at room temperature, cover slides were washed 3 times for 5 minutes with PBS/0.2% Tween. Secondary detection was done using a 1:200 dilution of a goat-anti human IgG-Alexa488 (Dianova). After 30 minutes at room temperature, slides were washed 3 times for 5 minutes and rinsed with water. Slides were mounted using DAPI prolonged mounting medium (FisherScientific).

#### Crystal structure determination of Fab-RBD complexes

The coding sequence for receptor binding domain (RBD; residues 319-541) of the SARS-CoV-2 spike (S) protein was synthesized and cloned into a customized pFastBac vector (Ekiert et al., 2011), which is designed to fuse an N-terminal gp67 signal peptide and C-terminal His_6_ tag to the target protein. To express the RBD protein, a recombinant bacmid DNA was generated from the sequencing-confirmed pFastBac construct using the Bac-to-Bac system (Life Technologies). Baculovirus was generated by transfecting purified bacmid DNA into Sf9 cells using FuGENE HD (Promega), and subsequently used to infect suspension cultures of High Five cells (Life Technologies) at a multiplicity of infection (MOI) of 5 to 10. Infected High Five cells were incubated at 28 °C with shaking at 110 rpm for 72 hours for protein expression. RBD protein that was secreted into the supernatant was then concentrated using a 10 kDa MW cutoff Centramate cassette (Pall Corporation). The RBD protein in the concentrate was purified by affinity chromatography using Ni-NTA resin (QIAGEN), followed by size exclusion chromatography on a HiLoad Superdex 200 pg column (GE Healthcare), and buffer exchanged into 20 mM Tris-HCl pH 7.4 and 150 mM NaCl using the same protocol as before (Yuan et al., 2020b). Fabs were expressed in ExpiCHO cells and purified using affinity and size exclusion chromatography. Residues in the elbow region of CV07-250 (^112^SSASTKG^118^) were mutated to ^112^FNQIKP^117^ to reduce elbow flexibility and facilitate crystal packing (Bailey et al., 2018). The Fab/RBD complexes were formed by mixing the two components in an equimolar ratio and incubating overnight at 4°C before setting-up crystal trials. The Fab/RBD complexes were screened for crystallization using 384 conditions of the JCSG Core Suite (QIAGEN) on our robotic CrystalMation system (Rigaku) at The Scripps Research Institute. Crystallization trials were set up for SARS-CoV-2 RBD in complex with a number of Fabs from this study, including CV07-250, CV07-270, CV07-283, CV07-287, CV07-209, CV07-222, CV07-262, CV38-113, and CV38-183, but only CV07-250/RBD and CV07-270/RBD produced diffraction quality crystals in complex with the SARS-CoV-2 RBD. CV07-250 and CV07-270 Fabs were expressed in ExpiCHO cells and purified using affinity and size exclusion chromatography. The Fab/RBD complexes were formed by mixing the two components in an equimolar ratio and incubating overnight at 4°C before setting-up crystal trials. The complexes of CV07-250/RBD and CV07-270/RBD were screened for crystallization at 20.0 and 12.0 mg/ml, respectively, using 384 conditions of the JCSG Core Suite (QIAGEN) on our robotic CrystalMation system (Rigaku) at The Scripps Research Institute. Crystals appeared after day 3, were harvested after day 7, and then flash-cooled in liquid nitrogen for X-ray diffraction experiments. Diffraction data were collected at cryogenic temperature (100 K) at Stanford Synchrotron Radiation Lightsource (SSRL) on the newly constructed Scripps/Stanford beamline 12-1 with a beam wavelength of 0.97946 Å and processed with HKL3000 (Minor et al., 2006). Diffraction data were collected from crystals grown in conditions: 0.085 M HEPES pH 7.5, 10% (v/v) ethylene glycol, 15% (v/v) glycerol, 8.5% (v/v) 2-propanol, 17% (w/v) polyethylene glycol 4000 for the CV07-250/RBD complex and 0.1 M sodium cacodylate pH 6.5, 0.2 M sodium chloride, 2 M ammonium sulfate, 15% (v/v) ethylene glycol for the CV07-270/RBD complex. The X-ray structures were solved by molecular replacement (MR) using PHASER (McCoy et al., 2007) with MR models for the RBD and CV07-270 Fab from PDB 6W41 (Yuan et al., 2020b) and 4FQH, respectively. The MR model for CV07-250 Fab was generated using Repertoire Builder (Schritt et al., 2019). Iterative model building and refinement were carried out in COOT (Emsley and Cowtan, 2004) and PHENIX (Adams et al., 2010), respectively. In the CV07-250 + RBD structure, residues 319-337, 357- 366, 371-374, 383- 396, 516-541 were not modeled due to paucity of electron density. The N and C-terminal regions are normally disordered in the SARS CoV-2 RBD structures. These flexible regions are not involved in any other contacts, including crystal lattice contacts, and are on the opposite side of the RBD to the epitope, which is well ordered. In the CV07-270 + RBD structure, Fab residues in a region of the heavy-chain constant domain also have greater mobility as commonly found in Fabs. Likewise, the N and C-terminal residues of 319-333 and 528-541 in both RBD molecules of the asymmetric unit are disordered. Epitope and paratope residues, as well as their interactions, were identified by accessing PDBePISA (Krissinel and Henrick, 2007) at the European Bioinformatics Institute (https://www.ebi.ac.uk/pdbe/prot_int/pistart.html).

#### Murine tissue reactivity screening

Preparations of brain, lung, heart, liver, kidney and gut from 8-12 weeks old C57BL/6J mice were frozen in −50°C 2-methylbutane, cut on a cryostat in 20 μm sections and mounted on glass slides. For tissue reactivity screening according to established protocols (Kreye et al., 2016), thawed unfixed tissue slices were rinsed with PBS then blocked with blocking solution (PBS supplemented with 2% Bovine Serum Albumin (Roth) and 5% Normal Goat Serum (Abcam)) for 1 hour at room temperature before incubation of mAbs at 5 μg/ml overnight at 4°C. After three PBS washing steps, goat anti-human IgG-Alexa Fluor 488 (Dianova, 109-545-003) diluted in blocking solution was applied for 2 hours at room temperature before additional three washes and mounting using DAPI-containing Fluoroshield. Staining was examined under an inverted fluorescence microscope (Olympus CKX41, Leica DMI6000) or confocal device (Leica TCS SL). For co-staining, tissue was processed as above, but sections were fixed with 4% PFA in PBS for 10 minutes at room temperature before blocking. For co-staining, the following antibodies were used: mouse Smooth Muscle Actin (clone 1A4, Agilent, 172 003), goat anti-mouse IgG-Alexa Fluor 594 (Dianova, 115-585-003). For nuclei staining DRAQ5 (abcam, ab108410) was used.

#### HEp2 cell assay

HEp-2 cell reactivity was investigated using the NOVA Lite HEp-2 ANA Kit (Inova Diagnostics) according to the manufacturer’s instructions using mAb containing culture supernatant (screening of all S1+ mAbs) or purified mAbs at 50 μg/ml (polyreactivity testing of CV07-200, CV07-209, CV07-222, CV07-255, CV07-270 and CV38-148) and examined under an inverted fluorescence microscope.

#### Polyreactivity screening ELISA

Purified mAbs were screened for reactivity against cardiolipin and beta-2 microglobulin at 50 μg/ml using routine laboratory ELISAs kindly performed by Christian Meisel (Labor Berlin).

#### Hamster model of SARS-CoV-2 infection

Virus stocks for animal experiments were prepared from the previously published SARS-CoV-2 München isolate (Wölfel et al., 2020). Viruses were propagated on Vero E6 cells (ATCC CRL-1586) in minimal essential medium (MEM; PAN Biotech) supplemented with 10% fetal bovine serum (PAN Biotech) 100 IU/ml Penicillin G and 100 μg/ml Streptomycin (Carl Roth). Stocks were stored at −80°C prior to experimental infections.

For the SARS-CoV-2 challenge experiments, hamsters were randomly distributed into three groups: In the first group (prophylaxis group), animals received an intraperitoneal (i.p.) injection of 18 mg per kg bodyweight of SARS-CoV-2 neutralizing mAb CV07-209 24 hours prior to infection. In the second and third group (treatment and control group, respectively), animals were given the identical mAb amount two hours after infection, either with 18 mg/kg of CV07-209 (treatment group) or with 20 mg/kg of non-reactive isotype-matched mGO53 (control group). Hamsters were infected intranasally with 1x10^5^ PFU SARS-CoV-2 diluted in minimal essential medium (MEM; PAN Biotech) to a final volume of 60 μl as previously described (Osterrieder et al., 2020).

On days 2, 5 and 13 post infection, three hamsters of each group were euthanized by exsanguination under general anesthesia employing a protocol developed specifically for hamsters and consisting of 0.15 mg/kg medetomidine, 2 mg/kg midazolam and 2.5 mg/kg butorphanol applied as a single intramuscular injection of 200 μl (Nakamura et al., 2017). Nasal washes, tracheal swabs, and lungs (left and right) were collected for histopathological examinations and/or virus titrations and RT-qPCR. Body weights were recorded daily and clinical signs of all animals were monitored twice daily throughout the experiment.

#### Histopathology and *in situ* hybridization

For histopathological examination and *in situ* hybridization (ISH) of lung tissues, the left lung lobe was carefully removed and immersed in fixative solution (4% formalin, pH 7.0) for 48 hours. Lungs were then embedded in paraffin and cut in 2 μm sections. For histopathology, slides were stained with hematoxylin and eosin (HE) after dewaxing in xylene and rehydration in decreasing ethanol concentrations. Lung sections were microscopically evaluated in a blinded fashion by board-certified veterinary pathologists to assess the character and severity of pathologic lesions using lung-specific inflammation scoring parameters (Dietert et al., 2017) as previously described for SARS-Cov2 infection in hamsters (Gruber et al., 2020; Osterrieder et al., 2020). These parameters included severity of interstitial pneumonia, immune cell infiltration by neutrophils, macrophages, and lymphocytes, bronchitis, epithelial necrosis of bronchi and alveoli, hyperplasia of bronchial epithelial cells and type II-alveolar epithelial cells, endothelialitis, perivascular lymphocytic cuffing, as well as alveolar edema and perivascular edema (Table S6). The following parameters were evaluated to assess three different scores: (1) the bronchitis score that includes severity of bronchial inflammation and epithelial cell necrosis of bronchi (Figures 6B and 6E), (2) the regeneration score including hyperplasia of bronchial epithelial cells and type-II-alveolar epithelial cells, and (3) the edema score including alveolar and perivascular edema (Figures 6B and 6E).

ISH was performed as reported previously (Erickson et al., 2020; Osterrieder et al., 2020) using the ViewRNA ISH Tissue Assay Kit (Invitrogen by Thermo Fisher Scientific) following the manufacturer’s instructions with the minor following adjustments. Probes for the detection of the Nucleoprotein (N) gene RNA of SARS-CoV-2 (NCBI database NC_045512.2, nucleotides 28,274 to 9,533, assay ID: VPNKRHM), and the murine housekeeping gene eukaryotic translation elongation factor-1α (EF1a; assay ID: VB1-14428-VT, Affymetrix, Inc.), that shares 95% sequence identity with the Syrian hamster, were designed. Lung sections (2 μm thickness) on adhesive glass slides were dewaxed in xylol and dehydrated in ethanol. Tissues were incubated at 95°C for 10 minutes with subsequent protease digestion for 20 minutes. Sections were fixed with 4% paraformaldehyde in PBS (Alfa Aesar, Thermo Fisher) and hybridized with the probes. Amplifier and label probe hybridizations were performed according to the manufacturer’s instructions using fast red as the chromogen, followed by counterstaining with hematoxylin for 45 s, washing in tap water for 5 minutes, and mounting with Roti®-Mount Fluor-Care DAPI (4, 6-diaminidino-2-phenylindole; Carl Roth). An irrelevant probe for the detection of pneumolysin was used as a control for sequence-specific binding. HE-stained and ISH slides were analyzed and images taken using a BX41 microscope (Olympus) with a DP80 Microscope Digital Camera and the cellSens Imaging Software, Version 1.18 (Olympus).

#### Virus titrations, RNA extractions and RT-qPCR

To determine virus titers from 25 mg lung tissue, tissue homogenates were serially diluted and titrated on Vero E6 cells in 12-well-plates. Three days later, cells were formalin-fixed, stained with crystal violet and plaques were counted. RNA was extracted from homogenized lungs, nasal washes and tracheal swabs using the innuPrep Virus DNA/RNA Kit (Analytik Jena) according to the manufacturer’s instructions. We quantified RNA using a one-step RT qPCR reaction with the NEB Luna Universal Probe One-Step RT-qPCR kit (New England Biolabs) by following the manufacturer’s instructions and by using previously published TaqMan primers and probes (SARS-CoV-2 E_Sarbeco and hamster RPL18) (Corman et al., 2020; Zivcec et al., 2011) on a StepOnePlus RealTime PCR System (Thermo Fisher Scientific). Primer concentrations were 400 nM for forward (E_Sarbeco_F; 5′-ACAGGTACGTTAATAGTTAATAGCGT-3′; RPL18_F: 5′-GTTTATGAGTCGCACTAACCG-3′) and reverse (E_Sarbeco_R; 5′-ATATTGCAGCAGTACGCACACA-3′; RPL18_R: 5′-TGTTCTCTCGGCCAGGAA-3′) primers, and 200 nM for the TaqMan Probe (E_Sarbeco_P: FAM-ACACTAGCCATCCTTACTGCGCTTCG-BBQ; RPL18_P YAK-TCTGTCCCTGTCCCGGATGATC-BBQ).

Detection of subgenomic RNA (sgRNA) was done using by oligonucleotides targeting the leader transcriptional regulatory sequence and by oligonucleotides targeting regions within the E gene, as described previously (Corman et al., 2020; Wölfel et al., 2020): The sgRNA RT-PCR assay used the Platinum SuperScript III RT-PCR-System with Platinum Taq DNA Polymerase (Thermo Fisher Scientific). A 25 μL reaction contained 5 μL of RNA, 12.5 μL of 2 × reaction buffer provided with the kit (containing 0.4 mM of each deoxyribont triphosphates (dNTP) and 3.2 mM magnesium sulfate), 1 μL of reverse transcriptase/Taq mixture from the kit, 1 μg of nonacetylated bovine serum albumin (Roche), and 0.4 μL of a 50 mM magnesium sulfate solution (Thermo Fisher Scientific). Primer concentrations were 400 nM for forward (F; 5′-CGATCTCTTGTAGATCTGTTCTC-3′) and reverse (R; 5′-ATATTGCAGCAGTACGCACACA-3′) primers, and 200 nM for the TaqMan Probe P; (FAM-ACACTAGCCATCCTTACTGCGCTTCG-BBQ). Thermal cycling involved 10 min at 50°C for reverse transcription, followed by 3 min at 95°C and 45 cycles of 10 s at 95°C, 15 s at 56°C, and 5 s at 72°C.

### Quantification and Statistical Analysis

All statistical tests were performed using GraphPad Prism, version 8.4. For comparison of SHM number (Figure S1D), a D’Agostino-Pearson normality test showed that the number of SHM was not normally distributed, therefore a Kruskal-Wallis test was used with posthoc Dunn’s multiple comparisons tests. For bodyweight changes from hamster experiments (Figure 5B) a D’Agostino-Pearson normality test revealed normal distribution, Thus, statistical significance of bodyweight changes from hamster experiments was tested using a mixed-effects model (two-way ANOVA) with posthoc Dunnett’s multiple comparisons test in comparison to control group. Statistical details can be found in the figure legends. For top 18 mAbs, EC_50_ and IC_50_ values (Figure 1; Table S3) were determined from non-linear regression models using Graph Pad Prism 8.4. Binding kinetics of mAbs to RBD were modeled from multi-cycle surface plasmon resonance measurements (Figure S4) using the Biacore T200 software, version 3.2.

## Supplemental Information

Document S1. Tables S1 and S3–S6

Table S2. Ig Sequence and Functional Screening Data of All Isolated mAbs, Related to Figures 1, S1, S2, and S3 For clonality analysis mAbs from an identical donor were identified as clones when sharing the same V and J gene on both heavy and light chain and showing similarities within amino acid sequences of both CDR3. S1 reactivity was determined as a normalized optical density (OD) measured by SARS-CoV-2-S1 ELISA, with values above 0.5 considered as positive. From S1+ mAbs specificity to the RBD was determined using an RBD ELISA and noted as negative (-), positive (+) or strongly positive (++), when detectable at 10 ng/ml. All strongly positive RBD+ mAbs were screened for neutralization of authentic SARS-CoV-2 (Figure 1A), from which the IC_50_ was estimated.
