# Supplementary material for: A Therapeutic Non-self-reactive SARS-CoV-2 Antibody Protects from Lung Pathology in a COVID-19 Hamster Model
Source: Cell. 2020 Nov 12;183(4):1058–1069.e19. doi: 10.1016/j.cell.2020.09.049 (PMC7510528; doi:10.1016/j.cell.2020.09.049)
Supplement: Document S1. Tables S1 and S3–S6 [file mmc1.pdf]

Cell, Volume *183*

Supplemental Information

A Therapeutic Non-self-reactive SARS-CoV-2 Antibody Protects from Lung Pathology in a COVID-19 Hamster Model

Jakob Kreye, S. Momsen Reincke, Hans-Christian Kornau, Elisa Sánchez-Sendin, Victor Max Corman, Hejun Liu, Meng Yuan, Nicholas C. Wu, Xueyong Zhu, Chang-Chun D. Lee, Jakob Trimpert, Markus Höltje, Kristina Dietert, Laura Stöffler, Niels von Wardenburg, Scott van Hoof, Marie A. Homeyer, Julius Hoffmann, Azza Abdelgawad, Achim D. Gruber, Luca D. Bertzbach, Daria Vladimirova, Lucie Y. Li, Paula Charlotte Barthel, Karl Skriner, Andreas C. Hocke, Stefan Hippenstiel, Martin Witzenrath, Norbert Suttorp, Florian Kurth, Christiana Franke, Matthias Endres, Dietmar Schmitz, Lara Maria Jeworowski, Anja Richter, Marie Luisa Schmidt, Tatjana Schwarz, Marcel Alexander Müller, Christian Drosten, Daniel Wendisch, Leif E. Sander, Nikolaus Osterrieder, Ian A. Wilson, and Harald Prüss

Table S1. Description of patient cohort. Related to Figures 1, S1.

**General**	**Patient ID** (gender, age)	**CV01** (m, 21)	**CV03** (m, 46)	**CV05** (m, 61)	**CV07** (m, 32)	**CV23** (f, 44)	**CV24** (m, 64)	**CV38** (m, 70)	**CV48** (m, 48)	**CV-X1** (m, 46)	**CV-X2** (m, 57)
Patient history	Pre-existing conditions	N	CVD, Crohn's disease	N	N	atopic dermatitis, hypthyreosis	CVD	CVD	N	N	N
	Smoking	N	N	N	Y	N	N	Y (10 PY)	N	Y (7 PY)	N
Symptoms	Pulmonary and general symptoms	fever, cough, emesis	fever, cough, dyspnea, emesis	fever, cough, dyspnea, superinfection	fever, cough, dyspnea, respiratory failure	fever	fever, cough, dyspnea, emesis	fever, dyspnea, renal failure, atrial fibrillation	fever, cough, dyspnea, diarrhea	fever, cough, dyspnea, respiratory failure, diarrhea	fever, cough, epistaxis
	Neurological symptoms	loss of smell, muscle twitches	loss of smell, mood impairment, paresthesia	N	loss of smell	n/a	loss of smell	N	N	n/a	loss of smell
Disease course	Date symptom onset	17.02.2020	29.02.2020	27.02.2020	08.03.2020	20.03.2020	13.03.2020	12.03.2020	14.03.2020	15.03.2020	18.03.2020
	Days until diagnosis / hospitalization	13 / 13	10 / 11	3 / 13	5 / 6	0 / 0	7 / 8	6 / 14	10 / 9	11 / 12	2 / -
	SARS-CoV-2 serum IgG at PBMC isolation	2.0 / 3.8	6.4	5.7	6.6	1.2	n/a	8.9	6.4	n/a	n/a
	Days in hospital / on ICU	34 / 0	14 / 0	9 / 0	15 / 2	34 / 0	8 / 2	15 / 0	17 / 2	15 / 6	0 / 0
	Oxygen demand / days ventilation	N / 0	N / 0	N / 0	Y / 0	N / 0	Y / 0	Y / 0	Y / 0	Y / 3	N / 0
	Medication	N	N	AB, ACT	N	N	AB, ACT	AB, ACT, APT, Digitoxin	AB, ACT, APT, HCQ	AB, HCQ	N
Follow Up	Outcome	discharged	discharged	discharged	discharged	discharged	discharged	discharged	discharged	discharged	discharged

PBMCs from ten COVID-19 patients were included in immunoglobulin repertoire studies. General information, patients' history, symptoms of SARS-CoV-2 infection, disease course and outcome are listed for each donor. SARS-CoV-2 IgG is given as a ratio of optical density from time point of PBMC isolation (± one day).

Abbreviations: m = male, f = female, Y = yes, N = no, n/a = not available, CVD = cardiovascular disease, PY = pack years, AB = antibiotics, ACT = anticoagulation therapy, APT = antiplatelet therapy, HCQ = hydroxychloroquine.

Table S3. Biophysical and functional characterization of the 18 most potent SARS-CoV-2 neutralizing mAbs. Related to Figures 1, 2, S4, S5.

mAb	EC_{50} (ng/ml)	K_a (s^{-1})	K_d ($M^{-1}s^{-1}$)	K_D (M)	IC_{50} (ng/ml)	ACE2 binding reduction (%)	Tissue reactivity
CV05-163	3.8	8.48 E+06	1.86 E-03	2.20 E-10	16.3	9.6	none
CV07-200	9.0	n/a	n/a	n/a	14.5	none	B / L*
CV07-209	4.1	2.40 E+07	1.44 E-04	6.00 E-12	3.1	73.9	none
CV07-222	5.5	2.98 E+07	2.07 E-04	6.97 E-12	7.8	36.9	B / L / C
CV07-250	8.7	4.05 E+06	2.28 E-04	5.64 E-11	3.5	59.9	none
CV07-255	4.8	n/a	n/a	n/a	14.5	51.6	L / H / C / K
CV07-262	12.1	4.27 E+06	3.37 E-04	7.90 E-11	7.1	25.5	none
CV07-270	14.2	n/a	n/a	n/a	82.3	7.5	L / H* / C
CV07-283	4.0	1.52 E+07	7.24 E-04	4.75 E-11	16.9	38.0	none
CV07-287	5.9	5.11 E+06	8.14 E-04	1.60 E-10	41.7	12.4	none
CV07-315	7.2	2.36 E+07	2.62 E-04	1.12 E-11	24.9	none	none
CV38-113	5.2	2.33 E+06	3.12 E-04	1.34 E-10	20.8	51.2	none
CV38-139	8.9	3.62 E+05	1.00 E-04	2.77 E-10	73.2	14.9	none
CV38-142	5.6	6.31 E+05	6.60 E-04	1.05 E-09	23.2	26.9	none
CV38-183	5.4	2.31 E+06	1.37 E-04	5.93 E-11	3.7	65.9	none
CV38-221	6.4	7.45 E+05	3.71 E-04	4.99 E-10	172.6	50.1	none
CV-X1-126	5.5	4.12 E+05	9.98 E-05	2.43 E-10	71.7	44.2	none
CV-X2-106	5.6	n/a	n/a	n/a	17.6	19.9	none

Abbreviations: EC_{50} = half-maximal effective concentration, K_a = association rate, K_d = dissociation rate, K_D = equilibrium dissociation constant, IC_{50} = half-maximal inhibitory concentration, B = brain, L = lung, H = heart, C = colon, K = kidney. An asterisk (*) indicates weak binding.

Table S4. X-ray data collection and refinement statistics. Related to Figures 3, 4.

	CV07-250 + RBD	CV07-270 + RBD
**Data collection**		
Beamline	SSRL 12-1	SSRL 12-1
Wavelength (Å)	0.97946	0.97946
Space group	$P\ 2_1\ 2_1\ 2_1$	C 1 2 1
Unit cell parameters		
a, b, c (Å)	68.0, 80.1, 153.7	157.0, 151.8, 66.0
α, β, γ (°)	90, 90, 90	90, 95.4, 90
Resolution (Å) [a]	50.0–2.55 (2.59–2.55)	50.0–2.70 (2.75–2.70)
Unique reflections [a]	27,890 (2,659)	41,624 (2,089)
Redundancy [a]	8.8 (6.5)	4.7 (3.6)
Completeness (%) [a]	99.6 (98.9)	90.8 (90.8)
$<I/\sigma_I>$ [a]	17.3 (1.1)	6.7 (1.1)
R_{sym} [b] (%) [a]	11.9 (94.6)	19.9 (80.6)
R_{pim} [b] (%) [a]	4.1 (38.9)	9.9 (50.5)
$CC_{1/2}$ [c] (%) [a]	99.5 (71.0)	97.6 (67.6)
**Refinement statistics**		
Resolution (Å)	43.2–2.55	49.7–2.72
Reflections (work)	27,883	39,568
Reflections (test)	2,656	2,055
R_{cryst} [d] / R_{free} [e] (%)	20.7/25.9	22.6/26.7
No. of atoms	4,525	9,233
Macromolecules	4,420	9,165
Glycans	28	28
Solvent	77	40
Average *B*-value ($Å^2$)	52	44
Macromolecules	52	44
Fab	46	42
RBD	66	46
Glycans	100	77
Solvent	49	57
Wilson *B*-value ($Å^2$)	51	39
**RMSD from ideal geometry**		
Bond length (Å)	0.005	0.007
Bond angle (°)	0.74	1.27
**Ramachandran statistics (%)**		
Favored	96.1	96.8
Outliers	0.2	0.0
PDB code	6XKQ	6XKP

[a] Numbers in parentheses refer to the highest resolution shell.
[b] $R_{sym} = \Sigma_{hkl} \Sigma_i | I_{hkl,i} - <I_{hkl}> | / \Sigma_{hkl} \Sigma_i I_{hkl,i}$ and $R_{pim} = \Sigma_{hkl} (1/(n-1))^{1/2} \Sigma_i | I_{hkl,i} - <I_{hkl}> | / \Sigma_{hkl} \Sigma_i I_{hkl,i}$, where $I_{hkl,i}$ is the scaled intensity of the ith measurement of reflection h, k, l, $<I_{hkl}>$ is the average intensity for that reflection, and *n* is the redundancy.
[c] $CC_{1/2}$ = Pearson correlation coefficient between two random half datasets.
[d] $R_{cryst} = \Sigma_{hkl} | F_o - F_c | / \Sigma_{hkl} | F_o | \times 100$, where F_o and F_c are the observed and calculated structure factors, respectively.
[e] R_{free} was calculated as for R_{cryst}, but on a test set comprising 5% of the data excluded from refinement.

Table S5. Hydrogen bonds and salt bridges identified at the antibody-RBD interface using the PISA program. Related to Figures 3, 4.

CV07-250	Distance (Å)	SARS-CoV-2 RBD
	**Hydrogen bonds**	
H:TYR98[N]	3.0	A:ALA475[O]
H:ASN31[ND2]	3.4	A:SER477[OG]
H:ASN100C[ND2]	3.8	A:GLY485[O]
H:ASN100C[ND2]	2.9	A:PHE486[O]
H:ASN97[ND2]	2.8	A:ASN487[OD1]
H:ASN97[ND2]	2.7	A:TYR489[OH]
H:TYR100B[N]	3.5	A:TYR489[OH]
H:ASP96[OD1]	3.0	A:SER477[N]
H:ASP96[OD2]	2.7	A:SER477[OG]
H:ASP96[OD2]	3.5	A:THR478[N]
H:ASP96[OD2]	2.9	A:THR478[OG1]
H:SER95[OG]	2.9	A:ASN487[ND2]
H:PHE100[O]	3.4	A:TYR489[OH]
L:SER27A[OG]	3.2	A:TYR505[OH]
L:HIS31[N]	3.1	A:GLN493[OE1]
L:SER67[OG]	3.5	A:GLY446[O]
L:GLY68[N]	3.1	A:GLY446[O]
L:GLY68[N]	3.1	A:TYR449[OH]
L:ASN69[N]	3.8	A:GLN498[OE1]
L:ASN69[ND2]	3.7	A:GLN498[OE1]
L:TYR49[OH]	3.5	A:PHE486[N]

CV07-270	Distance (Å)	SARS-CoV-2 RBD
	**Hydrogen bonds**	
H:TYR100B[OH]	2.8	A:SER349[OG]
H:SER98[N]	3.2	A:GLY447[O]
H:SER98[OG]	3.8	A:GLY447[O]
H:SER98[OG]	3.2	A:ASN448[OD1]
H:ARG100G[NH2]	2.9	A:GLU484[OE1]
H:ARG100G[NH1]	3.2	A:GLU484[OE2]
H:ARG100G[NH2]	3.0	A:GLU484[OE2]
H:THR28[OG1]	3.2	A:LYS444[NZ]
H:ASP31[OD1]	2.7	A:ARG346[NH1]
H:ASP31[OD2]	2.7	A:LYS444[NZ]
H:SER98[O]	2.7	A:TYR449[N]
H:SER98[O]	3.3	A:ASN450[N]
H:TYR100B[OH]	3.6	A:SER349[N]
	**Salt bridges**	
H:ARG100G[NH2]	2.9	A:GLU484[OE1]
H:ARG100G[NH1]	3.2	A:GLU484[OE2]
H:ARG100G[NH2]	3.0	A:GLU484[OE2]
H:ASP31[OD1]	2.7	A:ARG346[NH1]
H:ASP31[OD1]	3.4	A:ARG346[NE]
H:ASP31[OD2]	2.7	A:LYS444[NZ]

Table S6. Histopathological scoring of lung tissue from COVID-19 hamster model. Related to Figures 5, 6.

		pneumonia																
Animal number	Time point (dpi)	% Affected lung tissue	Degree of inflammation*	Lymphocytes	Macrophages	Neutrophils	Epithelial cell necrosis of bronchi[a]	Bronchial inflammation[a]	Hyperplasia of bronchial epithelia cells[b]	Alveolar epithelial cell necrosis	Hyperplasia type II alveolar epithelial cells[b]	Alveolar edema[c]	Perivascular edema[c]	Perivascular lymphocytic cuffing	Endotheliitis	[a] Bronchitis score	[b] Regeneration score	[c] Edema score
P1	3	<5	1.0	0.0	1.0	1.0	0.0	0.0	0.0	1.0	0.0	0.0	0.0	1.0	0.0	0.0	0.0	0.0
P2	3	<5	1.0	1.0	1.0	1.0	0.0	0.0	0.0	1.0	0.0	0.0	0.0	1.0	0.0	0.0	0.0	0.0
P3	3	5	1.0	1.0	1.0	1.0	0.0	0.0	0.0	1.0	0.0	0.0	0.0	0.0	0.0	0.0	0.0	0.0
T1	3	10	1.5	1.0	1.0	1.0	0.0	0.0	0.0	1.0	0.0	0.0	0.0	1.0	0.0	0.0	0.0	0.0
T2	3	25	3.0	2.5	2.0	3.5	3.5	4.0	0.0	3.0	2.0	1.5	0.0	2.0	2.0	3.8	1.0	0.8
T3	3	15	2.0	2.0	2.0	2.0	0.0	0.0	0.0	2.0	1.0	0.0	1.0	1.0	0.0	0.0	0.5	0.5
C1	3	15	2.5	2.0	2.0	3.0	2,5	3.0	0.0	3.0	1.0	2.0	1.0	1.0	2.0	3.0	0.5	1.5
C2	3	20	2.5	2.5	2.0	2.5	2,5	3.0	0.0	2.0	1.0	0.0	3.0	1.5	2.0	3.0	0.5	1.5
C3	3	20	2.5	2.0	2,5	2,5	2.0	2.0	0.0	3.0	2.0	0.0	2.0	1.0	2.0	2.0	1.0	1.0
P4	5	5-10	2.0	2.0	2.0	1.5	0.0	0.0	0.0	2.0	2.0	0.0	1.5	1.0	0.0	0.0	1.0	0.8
P5	5	<5	1.0	1.0	1.0	1.0	0.0	0.0	0.0	1.0	0.0	0.0	1.0	1.0	0.0	0.0	0.0	0.5
P6	5	15	2.0	2.0	2.0	2.0	0.0	0.0	0.0	2.0	2.0	0.0	0.0	2.0	0.0	0.0	1.0	0.0
T4	5	5-10	2.0	2.0	1.5	1.0	0.0	0.0	0.0	1.0	2.0	0.0	0.0	2.0	0.0	0.0	1.0	0.0
T5	5	25	3.0	3.0	2.0	3.0	2.0	2.0	0.0	3.0	3.0	2.0	2.0	3.0	3.0	2.0	1.5	2.0
T6	5	0	0.0	0.0	0.0	0.0	0.0	0.0	0.0	0.0	0.0	0.0	0.0	0.0	0.0	0.0	0.0	0.0
C4	5	80	4.0	4.0	3.0	3.0	3.0	3.0	3.0	3.0	4.0	3.0	3.5	3.0	3.5	3.0	3.5	3.3
C5	5	15-20	2.5	3.0	2.5	2.0	2.0	2.0	1.5	2.0	2.0	2.5	0.0	3.0	2.0	2.0	1.8	1.3
C6	5	50	4.0	3.5	3.0	3.0	3.0	3.0	4.0	2.5	4.0	3.5	4.0	3.5	3.0	3.0	4.0	3.8
P7	13	20	2.0	2.0	2.0	1.0	0.0	0.0	0.0	1.5	0.0	1.5	0.0	1.5	0.0	0.0	0.8	0.8
P8	13	30	2.0	2.0	2.0	2.0	0.0	0.0	0.0	1.5	0.0	0.0	0.0	2.0	0.0	0.0	1.0	0.0
P9	13	30	2.0	2.0	2.0	2.0	0.0	1.0	1.0	1.5	0.0	0.0	0.0	2.0	0.0	0.5	1.5	0.0
T7	13	15	2.0	2.0	2.0	1.5	0.0	0.0	0.0	1.5	0.0	0.0	0.0	2.0	0.0	0.0	1.0	0.0
T8	13	5	1.0	1.5	1.0	1.0	0.0	0.0	0.0	0.0	0.0	0.0	0.0	1.0	0.0	0.0	0.5	0.0
T9	13	20	1.5	1.5	1.0	1.5	0.0	0.0	0.0	1.0	0.0	0.0	0.0	1.5	0.0	0.0	0.8	0.0
C7	13	5	1.0	1.0	1.0	1.0	0.0	0.0	0.0	0.0	0.0	0.0	0.0	1.5	0.0	0.0	0.8	0.0
C8	13	40	2.0	2.0	2.0	2.0	0.0	1.0	1.0	1.5	0.0	0.0	1.5	2.0	0.0	0.5	1.5	0.0
C9	13	10	1.0	1.0	1.0	1.0	0.0	1.0	0.0	0.0	0.0	0.0	0.0	1.5	0.0	0.5	0.8	0.0

Histopathological scoring of formalin-fixed paraffin-embedded lung tissue from hamsters of experimental groups as abbreviated (P = prophylactic, T = therapeutic, C = control) at indicated days post infection (dpi). Degree of inflammation (*) is scaled as (1) minimal, (2) mild, (3) moderate or (4) severe. For all other parameters rating refers to occurrence rate from (1) sporadic, (2) mild, (3) moderate to (4) severe. Scores were assessed as means from parameters with corresponding letters as indicated.
